# Effects of Individual and Combined Water, Sanitation, Handwashing, and Nutritional Interventions on Child Respiratory Infections in Rural Kenya: A Cluster-Randomized Controlled Trial

**DOI:** 10.4269/ajtmh.19-0779

**Published:** 2020-03-30

**Authors:** Jenna Swarthout, Pavani K. Ram, Charles D. Arnold, Holly N. Dentz, Benjamin F. Arnold, Stephen Kalungu, Audrie Lin, Sammy M. Njenga, Christine P. Stewart, John M. Colford, Clair Null, Amy J. Pickering

**Affiliations:** 1 Civil and Environmental Engineering, Tufts University, Medford, Massachusetts;; 2 Epidemiology and Environmental Health, University at Buffalo, Buffalo, New York;; 3 Department of Nutrition, University of California, Davis, Davis, California;; 4 Francis I. Proctor Foundation, University of California, San Francisco, California;; 5 Innovations for Poverty Action, Nairobi, Kenya;; 6 Department of Epidemiology and Biostatistics, University of California, Berkeley, Berkeley, California;; 7 Kenya Medical Research Institute, Nairobi, Kenya;; 8 Center for International Policy Research and Evaluation, Mathematica Policy Research, Washington, District of Columbia

## Abstract

Poor nutrition and hand hygiene are risk factors for acute respiratory infections (ARIs). Safe drinking water and sanitation can reduce exposure to pathogens and encourage healthy immune responses, reducing the risk of ARIs. Within a trial assessing impacts of water, sanitation, and handwashing (WASH), and nutritional interventions, we evaluated effects on ARIs. The WASH Benefits cluster-randomized trial enrolled pregnant women from Kenyan villages and evaluated health outcomes in children born to enrolled mothers 1 and 2 years after intervention delivery. Geographically adjacent clusters were block-randomized into a passive control (no promotional visits), a double-sized active control (monthly visits to measure mid–upper arm circumference), and six intervention groups: chlorinated drinking water (W), improved sanitation (S), handwashing with soap (H), combined WSH, improved nutrition (N) through counseling and lipid-based nutrient supplementation (LNS), and combined WSHN. The main outcome was the prevalence of ARI symptoms (cough, panting, wheezing, or difficulty breathing) in children younger than 3 years. Masking participants was not possible. Analyses were intention-to-treat. Between November 2012 and May 2014, 702 clusters were enrolled, including 6,960 (year 1) and 7,088 (year 2) children with ARI data. The cluster-level intra-cluster correlation coefficient for ARIs was 0.026 across both years. Water, sanitation, and handwashing interventions with behavior change messaging did not reduce ARIs. Nutrition counseling and LNS modestly reduced ARI symptoms compared with controls in year 1 [prevalence ratio (PR): 0.87, 95% confidence interval (CI): 0.77–0.99], but no effect in the combined WSHN group weakens this finding.

## INTRODUCTION

Acute respiratory infections (ARIs) are a leading cause of morbidity and mortality in children younger than 5 years.^1^ In 2016, the WHO estimated that ARIs caused 14.4% (> 10,000) of under-five deaths in Kenya.^2^ Crowding, poor air quality, low income, low birth weight, malnutrition, inadequate breastfeeding, and lack of handwashing are potential risk factors for ARIs and pneumonia, an infection of the lung alveoli.^3–7^

Malnutrition mediates the ability of respiratory pathogens to colonize human hosts by compromising the immune system.^7^ Exclusive breastfeeding from 0 to 6 months provides infants with essential nutrients and antibodies, promoting healthy immune system development.^8^ A recent systematic review and meta-analysis of observational studies found that severe malnutrition and inadequate breastfeeding practices were associated with increased odds of acute lower respiratory illness mortality.^6^ A 2013 systematic review and meta-analysis reported that breastfeeding, defined heterogeneously across studies, reduced the incidence or prevalence of lower respiratory infections by 32% (95% CI: 23–40%) in children younger than 2 years.^9^ Evidence for preventing ARIs with nutrient supplementation is inconclusive. A recent Cochrane review identified three studies examining the impacts of lipid-based nutrient supplementation (LNS) on respiratory illnesses, none of which demonstrated differences between intervention and control groups.^10^ A cluster-randomized effectiveness trial found that LNS might reduce ARIs among infants in Bangladesh but yielded inconsistent results.^11^

Handwashing can reduce ARIs by interrupting the transmission of respiratory pathogens. A recent systematic review on randomized controlled trials conducted in low- and middle-income countries found strong evidence of hygiene interventions (including education and infrastructure) reducing caregiver-reported ARI symptoms in children living in urban households, but no effects were observed in rural households.^12^ A cluster-randomized controlled trial in urban Bangladesh, however, did not detect impacts of handwashing and water treatment promotion and hardware provision on reported respiratory illness.^13^ This finding was likely explained by moderate adherence to the handwashing intervention because reported respiratory illness was 18% lower (95% CI: 2–31%) among people who had soap and water present at the handwashing station than those who did not.^13^ The effectiveness of handwashing on ARIs, therefore, largely depends on the specific setting, intervention targets, and intervention adherence.^12^

Previous work demonstrates that children who suffer from severe, persistent, or increased durations of diarrhea are at a higher risk for ARIs.^14–16^ Many risk factors for ARIs and diarrhea overlap, including malnutrition.^17^ Associations between malnutrition, reduced immunity, and infections are well documented.^18^ Reducing diarrhea or exposure to fecal pathogens through drinking water quality or sanitation improvements could combat micronutrient loss and weakened immune system response. There is observational evidence of the effects of sanitation and drinking water quality on ARIs. A secondary analysis among control participants from a randomized trial in rural Bangladesh found that sharing a latrine with other households increased the likelihood of household contacts developing influenza-like illness (defined as fever in children aged < 5 years and fever with cough or sore throat in those aged ≥ 5 years) from an index case.^19^ A matched, control study in Mali found that the odds of children reporting having had symptoms of respiratory infections in the past week were 25% lower for those attending schools with a comprehensive water, sanitation, and hygiene program than those attending comparison schools.^20^

Because of overlapping risk factors and comorbidity between ARIs and diarrhea,^14^ a comprehensive nutrition intervention (i.e., promoting adequate prenatal nutrition; early initiation, exclusivity, and continuation of breastfeeding; appropriate complementary feeding; and providing nutrient supplements) combined with improved water quality, sanitation, and handwashing could reduce ARIs more than any of the components alone. In this article, we report the effects of the individual and combined water, sanitation, and handwashing (WASH), and nutritional interventions in the WASH Benefits trial on ARI symptoms, a prespecified tertiary outcome, in children younger than 3 years in rural Kenya. Intervention effects on the trial’s primary outcomes, length-for-age Z-scores (LAZ), and diarrhea prevalence were published elsewhere.^21^

## MATERIALS AND METHODS

### Study design.

The Kenya WASH Benefits study was a cluster-randomized trial conducted in rural villages in Bungoma, Kakamega, and Vihiga counties in western Kenya. Study design details for the Kenyan study and accompanying trial in Bangladesh were previously published.^21,22^ Villages were eligible for the study if they were rural, primarily relied on communal water sources and unimproved sanitation facilities, and were not enrolled in ongoing WASH or nutrition programs. Households were eligible if there was a pregnant woman in her second or third trimester who had planned to reside in her current residence for the next 2 years and could speak Kiswahili, Luhya, or English. Study clusters included up to three adjoining villages with at least six eligible pregnant women. Children born of these pregnancies (including twins) were defined as index children. Mothers provided written informed consent for their children and themselves.

Village clusters comprising 12 enrolled households, on average, were each randomized by blocks (groups of nine geographically adjacent clusters) into eight groups—passive control; active control; chlorinated drinking water; improved sanitation; handwashing with soap; combined WASH interventions; improved nutrition through infant and young child feeding counseling and LNS; and combined WASH and nutritional interventions—using a random number generator with a reproducible seed at the University of California, Berkeley. Community health promoters visited households monthly to measure child mid–upper arm circumferences in the randomized groups, excluding the passive control group. The purpose of the passive control group was to differentiate effects of household interaction with promoters from those of the interventions. In the six intervention groups, promoters delivered intervention-specific behavior change messaging, helped troubleshoot problems with hardware, and replenished supplies of chlorine, soap, and nutrient supplements. Masking participants was not possible because of the nature of the interventions, and participants were informed of their treatment assignment after baseline data collection. The data collection team was not informed of the treatment assignments but might have inferred assignments if they observed intervention materials in study communities. Investigators remained blinded to the treatment assignments until statistical analyses were replicated.

The WASH Benefits trial is registered at ClinicalTrials.gov, number NCT01704105. The trial protocol was approved by the Committee for the Protection of Human Subjects at the University of California, Berkeley (protocol number, 2011-09-3654), Institutional Review Board at Stanford University (IRB-23310), and Scientific and Ethics Review Unit at the Kenya Medical Research Institute (protocol number, SSC-2271). Innovations for Poverty Action was responsible for participant enrollment, intervention delivery, and data collection.

### Procedures.

Details of the trial implementation will be published elsewhere (https://osf.io/26r59/).^21^ Community health promoters were nominated by their local communities and trained to provide intervention-specific behavior change messaging, instructions on hardware use, and consumable supplies (chlorine, soap, and nutrient supplements). In intervention groups, promoters engaged caregivers of index children, and other compound members, through intervention-specific key messages, visual aids, interactive activities, and provision of hardware or products. Based on a literature review, a theory-based approach,^23,24^ formative research, and the WASH Benefits pilot randomized controlled trial,^25^ the developed behavior change messages focused on themes of nurture, aspiration, and self-efficacy. Interventions considered convenience and cultural norms to encourage adherence.

In water quality intervention groups, promoters advocated drinking water treatment with sodium hypochlorite using chlorine dispensers installed at communal water source collection points or bottled chlorine provided to households in study compounds. Promoters used chlorine test strips during monthly visits to determine stored water chlorine concentrations; negative results invoked discussions with households to address barriers to chlorination.

In sanitation groups, existing latrines were improved by installing plastic slabs with tight-fitting lids. Households without access to a latrine or access to a poor latrine were provided new latrines with plastic slabs and lids. Promoters advocated use of improved latrines for defecation and safe disposal of children’s and animals’ feces. All households in study compounds were provided plastic potties for each child younger than 3 years and sani-scoops with paddles for feces removal.

In handwashing groups, promoters advocated handwashing with soap before preparing food and after defecating (including assisting a child). Households were provided with two handwashing stations, one each near the food preparation area and the latrine. Stations were constructed with two-foot pedal-operated jerricans that dispensed soapy and rinse water. Households provided rinse water, but promoters added pieces of bar soap to the soapy water container every 3 months. Promoters helped participants identify compound members to refill tippy taps and manage barriers to use, such as running out of soap.

In nutrition groups, promoters delivered key messages for maternal, infant, and young child feeding around dietary diversity during pregnancy and lactation; early initiation of breastfeeding; exclusive breastfeeding from 0 to 6 months and continued breastfeeding through 24 months; introduction of appropriate and diverse complementary foods at 6 months; feeding frequency; and feeding during illness. Index children and siblings aged between 6 and 24 months were provided with two 10-g sachets per day of LNS (Nutriset, Malaunay, France), and caregivers were instructed to mix LNS into complementary foods twice a day.

After consent and enrollment, a baseline questionnaire was administered to collect data on household demographics; socioeconomics; water, sanitation, and hygiene behaviors; and food insecurity using the Household Hunger Scale.^26^ One and two years after intervention delivery, data were collected on adherence to interventions and child health outcomes (including ARI symptoms). Respiratory infection symptoms were collected for all children younger than 3 years in study households. The mean ages of index children and siblings younger than 3 years were 14.2 months (SD: 6.77 months) and 22.9 months (SD: 5.70 months) at years 1 and 2, respectively.

### Outcomes.

The primary outcome in this study is ARI symptoms—defined as having caregiver-reported cough or difficulty breathing, including panting or wheezing, within 7 days before the interview—in children younger than 3 years. Prespecified secondary outcomes in this study include difficulty breathing, including panting or wheezing, in the past 7 days (a more specific indicator of respiratory infection than a cough alone); ARI symptoms presenting with fever in the past 7 days (a potentially more severe infection); and enumerator-observed runny nose (an objective outcome). Enumerator-observed runny nose was a rare outcome, so caregiver-reported runny nose was examined in a post hoc analysis.

### Statistical analysis.

The sample size calculation for the main trial is described elsewhere.^21^ Sample size was chosen to detect a difference of 0.15 in LAZ and relative risk of diarrhea of ≤ 0.7 for comparing any intervention with the double-sized active control group; both calculations assume a type I error (α) of 0.05, power (1−β) of 0.8, and 10% loss to follow-up after the baseline.

Two masked researchers independently replicated the analyses following the prespecified analysis plan (https://osf.io/jre7x). We considered the intention-to-treat, unadjusted differences between each intervention group and a control group as our primary inference. The passive and active control groups were combined (hereby referred to as the control group); there were no differences in the prevalence of ARI symptoms between the two groups (data not presented). We compared outcomes in the combined water, sanitation, and handwashing group (WSH) to the individual intervention groups and the combined water, sanitation, handwashing, and nutrition group (WSHN) to the 1) nutrition group and 2) WSH group. We combined data from years 1 and 2 for the primary analyses. We conducted secondary analyses with year 1 data only because adherence to water treatment and handwashing interventions was higher.^22^ We used generalized linear models with robust standard errors and fixed effects for geographically pair-matched clusters. No adjustments for multiplicity were applied. We assessed effect modification of interventions on ARI symptoms for the following prespecified characteristics: index child status, child gender, and malaria seasonality. The peaks in ARI and pneumonia cases have been observed during malaria seasons, which occur shortly after wet seasons, in rural Kenya.^27^ We defined two malaria seasons: January–February and June–August. The sample size of non-index children (older siblings and children born during the study) was small, so results for the index child status subgroup analysis will not be presented; 10% (1,347/8,508) of children with ARI symptom data were non-index children. Analyses were done with Stata (version 14.2, StataCorp LLC, College Station, TX) and R (version 3.3.2, The R Foundation, Vienna, Austria).

After we unmasked the prespecified analyses’ results, we performed a post hoc analysis to interpret the main findings and examine whether intervention uptake differed between the single nutrition and combined WSHN arms. Previously, we reported that the prevalence of achieving minimum dietary diversity, defined as consuming at least four of seven key food groups (grains or tubers, legumes or nuts, dairy products, animal flesh foods, eggs, vitamin A–rich fruits and vegetables, and other fruits and vegetables) in the past 24 hours, was higher in the nutrition group than in the control group in the WASH Benefits trial but not when nutrition interventions were combined with improved water quality, sanitation, and handwashing.^28^ To test whether this difference persisted in the population of index children with ARI symptom data, we used generalized linear models (with robust standard errors and fixed effects for clusters) to compare the prevalence of achieving minimum dietary diversity in the nutrition versus WSHN group. We examined differences between the nutrition and WSHN groups in breastfeeding rates for index children for three indicators: any breastfeeding in the past 24 hours, early initiation of breastfeeding (i.e., caregiver reported putting the child to the breast immediately or within the first hour after birth), and exclusive breastfeeding for 6 months (i.e., caregiver reported exclusive breastfeeding for 6 months, corrected by the reported age of cessation and complementary foods eaten).

## RESULTS

**Figure 1.**
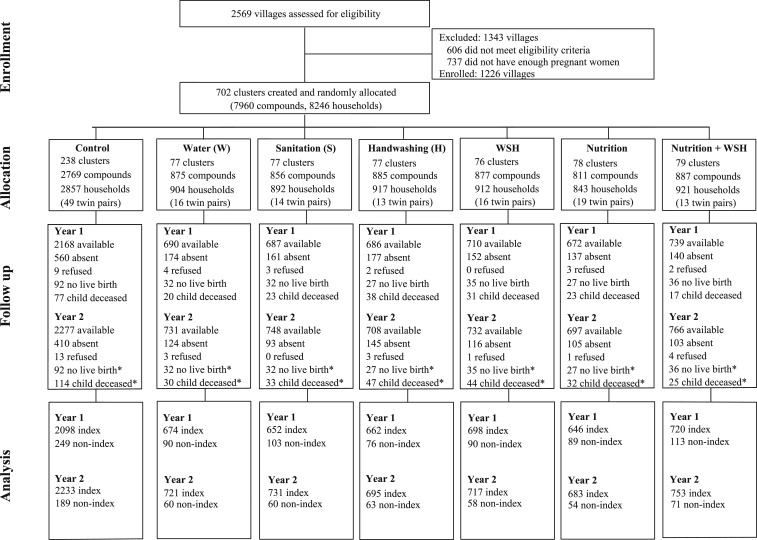
Trial profile and analysis population for acute respiratory illness outcome.

Two thousand five hundred sixty-nine villages were assessed for eligibility, of which 1,343 were excluded based on the village-level eligibility criteria (Figure 1). Between November 27, 2012 and May 21, 2014, 8,246 pregnant women residing in 702 clusters were enrolled. Of them, 281 women did not have a live birth and 140 women delivered twins. No clusters were lost to follow-up, but 2,212 households with 2,279 children were lost to follow-up by year 2. Losses to follow-up were balanced across groups. Acute respiratory infection symptom data were collected from 78% (6,150/7,876) of the surviving index children at year 1 and 84% (6,533/7,780) at year 2. Acute respiratory infection data were available for 810 and 555 non-index children at years 1 and 2, respectively.

Baseline characteristics were similar for households across groups (Table 1) and compared with those lost to follow-up (Supplemental Material). Most households had access to a protected drinking water source and latrine, but less than 17% (1,116/6,621) had access to a latrine with a slab or ventilation pipe. Open defecation was common in children and rare among adults. Less than 6% (413/7,045) of households had soap and water available at a handwashing station. The prevalence of moderate-to-severe household hunger was < 11% (736/7,033).

**Table 1:** Baseline household characteristics for children with acute respiratory infection symptom data

Number of households	Control (*N* = 2,432)	Water treatment (*N* = 770)	Sanitation (*N* = 782)	Handwashing (*N* = 755)	Water + sanitation + handwashing (*N* = 779)	Nutrition (*N* = 731)	Nutrition + water + sanitation + handwashing (*N* = 808)
Maternal							
Age (years)	26 (6)	27 (6)	26 (6)	26 (6)	26 (6)	26 (6)	26 (6)
Completed primary school, *n* (%)	1,158 (48)	376 (49)	379 (48)	333 (44)	359 (46)	357 (49)	383 (48)
Paternal, *n* (%)							
Completed primary school	1,398 (62)	452 (63)	434 (60)	418 (60)	456 (62)	437 (64)	465 (62)
Works in agriculture	980 (42)	331 (45)	322 (43)	308 (43)	329 (44)	308 (45)	331 (43)
Household							
Number of persons	5 (2)	5 (2)	5 (2)	5 (2)	5 (2)	5 (2)	5 (2)
Has electricity, *n* (%)	152 (6)	50 (7)	66 (8)	56 (7)	58 (7)	57 (8)	60 (7)
Has a cement floor, *n* (%)	134 (6)	63 (8)	43 (5)	29 (4)	43 (6)	38 (5)	51 (6)
Drinking water, *n* (%)							
Has protected primary drinking water source*	1,825 (75)	576 (75)	596 (76)	579 (77)	534 (69)	523 (72)	615 (76)
Stored water observed at home	1,984 (82)	626 (82)	643 (82)	628 (84)	624 (80)	596 (82)	659 (82)
Reported treating currently stored water	251 (13)	72 (12)	84 (13)	73 (12)	86 (14)	70 (12)	92 (14)
Sanitation							
Daily defecating in the open, *n* (%)							
Children aged 3 to < 8 years	197 (12)	63 (12)	62 (13)	63 (13)	66 (13)	68 (14)	68 (12)
Children aged 0 to < 3 years	1,034 (78)	331 (81)	331 (75)	299 (76)	344 (77)	317 (78)	350 (78)
Latrine, *n* (%)							
Owned by compound	2,001 (83)	641 (83)	631 (81)	621 (83)	652 (84)	614 (84)	671 (83)
Has access to latrine with a slab or ventilation pipe	391 (17)	124 (17)	116 (16)	126 (18)	127 (17)	105 (15)	127 (17)
Visible stool on the slab or floor	1,046 (47)	346 (49)	334 (47)	345 (50)	384 (53)	337 (49)	368 (49)
Has a potty	56 (2)	17 (2)	14 (2)	22 (3)	22 (3)	9 (1)	17 (2)
Human feces observed in the compound	207 (9)	54 (7)	67 (9)	69 (9)	65 (8)	63 (9)	79 (10)
Handwashing, *n* (%)							
Handwashing station has water and soap	129 (5)	51 (7)	38 (5)	41 (5)	58 (7)	49 (7)	47 (6)
Nutrition, *n* (%)							
Moderate to severe household hunger†	247 (10)	90 (12)	73 (9)	72 (10)	83 (11)	83 (11)	88 (11)

Data are *n* (%) or mean (SD). Percentages were calculated from smaller denominators than those shown at the top of the table for all variables because of missing values.

* Protected water sources include borewells, protected springs, protected dug wells, rainwater collection, and piped water into the home or yard/plot.

† Assessed by the Household Hunger Scale.

Roughly 74% (2,749/3,690) of households were visited by a community health promoter in the past month at year 1, but the frequency decreased below 37% (1,999/5,466) at year 2 (Supplemental Material). In water groups, 42% (499/1,174) and 21% (397/1,873) of households had detectable total chlorine in stored drinking water in years 1 and 2, respectively. In sanitation groups, > 80% of households had access to a latrine with a slab or ventilation pipe in years 1 and 2. In handwashing groups, 77% (1,156/1,490) of households had water and soap available at a handwashing station at year 1 and 21% (445/2,086) had handwashing materials at year 2. In nutrition groups, caregiver-reported consumption of LNS remained high; 95% (10,263/10,794) and 115% (7,612/6,608) of expected sachets (reported number of sachets consumed in the past week divided by the expected 14 sachets for index children aged between 6 and 24 months) were consumed at years 1 and 2, respectively.

**Figure 2.**
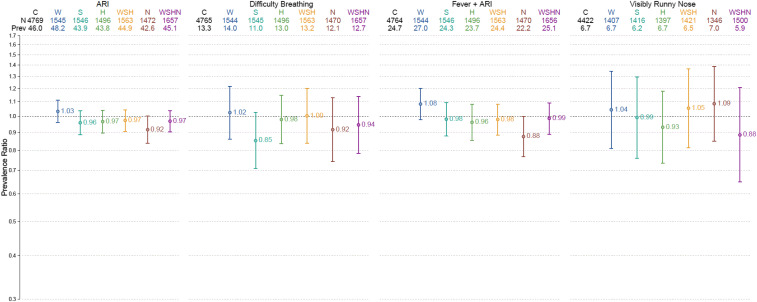
Respiratory outcome prevalence ratios by treatment status, years 1 and 2 combined. This figure appears in color at www.ajtmh.org.

**Figure 3.**
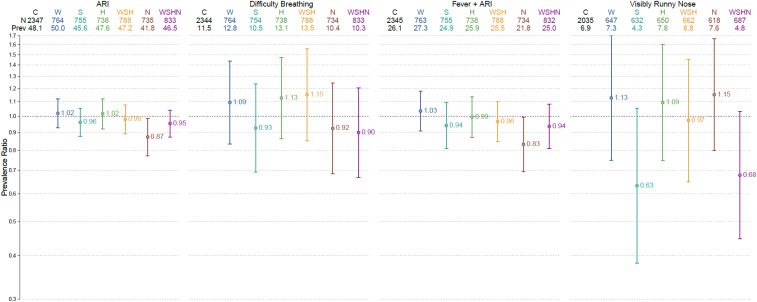
Respiratory outcome prevalence ratios by treatment status and year 1 only. This figure appears in color at www.ajtmh.org.

Acute respiratory infection symptom prevalence in the control group was 46% (2,193/4,769) in years 1 and 2 combined (Figure 2, Table 2). The intra-cluster correlation coefficient for ARI symptoms was 0.026 at the cluster level across both years. In the year 1–only analysis, ARI symptom prevalence in the nutrition group was 13% lower than in the control group (PR: 0.87, 95% CI: 0.77–0.99) (Figure 3, Table 2). Results were similar in years 1 and 2 combined, albeit marginally significant (PR: 0.92, 95% CI: 0.84–1.00) (Figure 2, Table 2). There were no other differences in ARI symptoms across intervention groups.

**Table 2:** Unadjusted effects of interventions on prevalence of respiratory outcomes, interventions vs. control

	Years 1 and 2 combined				Year 1 only			
Outcome, arm	*N*	Prevalence (%)	Prevalence ratio (95% CI)	*P*-value	*N*	Prevalence (%)	Prevalence ratio (95% CI)	*P*-value
ARI*								
Control	4,769	46.0	–	–	2,347	48.1	–	–
Water	1,545	48.2	1.03 (0.96–1.11)	0.395	764	50.0	1.02 (0.93–1.12)	0.703
Sanitation	1,546	43.9	0.96 (0.89–1.04)	0.289	755	45.6	0.96 (0.88–1.06)	0.407
Handwashing	1,496	43.8	0.97 (0.90–1.04)	0.351	738	47.6	1.02 (0.92–1.12)	0.768
Nutrition	1,472	42.6	0.92 (0.84–1.00)	0.054	735	41.8	0.87 (0.77–0.99)	0.029
WSH	1,563	44.9	0.97 (0.91–1.04)	0.439	788	47.2	0.98 (0.89–1.08)	0.672
WSHN	1,657	45.1	0.97 (0.90–1.04)	0.364	833	46.5	0.95 (0.87–1.04)	0.282
Panting, wheezing, or difficulty breathing								
Control	4,765	13.3	–	–	2,344	11.5	–	–
Water	1,544	14.0	1.02 (0.86–1.22)	0.787	764	12.8	1.09 (0.83–1.44)	0.520
Sanitation	1,545	11.0	0.85 (0.71–1.02)	0.089	754	10.5	0.93 (0.69–1.24)	0.602
Handwashing	1,496	13.0	0.98 (0.83–1.15)	0.786	738	13.1	1.13 (0.86–1.47)	0.380
Nutrition	1,470	12.1	0.92 (0.74–1.13)	0.408	734	10.4	0.92 (0.69–1.24)	0.600
WSH	1,563	13.2	1.00 (0.84–1.20)	0.976	788	13.5	1.15 (0.85–1.56)	0.362
WSHN	1,657	12.7	0.94 (0.78–1.14)	0.546	833	10.3	0.90 (0.67–1.20)	0.471
Fever and ARIs								
Control	4,764	24.7	–	–	2,345	26.1%	–	–
Water	1,544	27.0	1.08 (0.98–1.20)	0.125	763	27.3%	1.03 (0.91–1.18)	0.622
Sanitation	1,546	24.3	0.98 (0.88–1.09)	0.717	755	24.9%	0.94 (0.81–1.09)	0.423
Handwashing	1,496	23.7	0.96 (0.85–1.08)	0.511	738	25.9%	0.99 (0.87–1.14)	0.937
Nutrition	1,470	22.2	0.88 (0.77–1.00)	0.050	734	21.8%	0.83 (0.70–0.99)	0.041
WSH	1,563	24.4	0.98 (0.88–1.08)	0.676	788	25.5%	0.96 (0.85–1.10)	0.591
WSHN	1,656	25.1	0.99 (0.89–1.09)	0.777	832	25.0%	0.94 (0.81–1.08)	0.370
Visibly runny nose								
Control	4,422	6.7	–	–	2,035	6.9%	–	–
Water	1,407	6.7	1.04 (0.81–1.35)	0.745	647	7.3%	1.13 (0.75–1.70)	0.566
Sanitation	1,416	6.2	0.99 (0.76–1.30)	0.952	632	4.3%	0.63 (0.38–1.05)	0.078
Handwashing	1,397	6.7	0.93 (0.73–1.18)	0.546	650	7.8%	1.09 (0.75–1.60)	0.646
Nutrition	1,346	7.0	1.09 (0.85–1.39)	0.514	618	7.6%	1.15 (0.80–1.67)	0.451
WSH	1,421	6.5	1.05 (0.81–1.37)	0.694	662	6.8%	0.97 (0.65–1.45)	0.890
WSHN	1,500	5.9	0.88 (0.65–1.21)	0.439	687	4.8%	0.68 (0.45–1.03)	0.069

ARI = acute respiratory infection.

* An ARI was defined as caregiver-reported cough or difficulty breathing, including panting and wheezing, within the past 7 days before the interview.

The control group prevalence of difficulty breathing, ARI symptoms presenting with fever, visibly runny nose, and caregiver-reported runny nose was 13% (633/4,765), 25% (1,179/4,764), 7% (297/4,422), and 60% (2,885/4,769), respectively, in both years combined. The prevalence of ARI symptoms presenting with fever was 17% lower in the nutrition group than in the control group in year 1 (PR: 0.83, 95% CI: 0.70–0.99) and marginally lower when years 1 and 2 were combined (PR: 0.88, 95% CI: 0.77–1.00) (Figures 2 and 3, Table 2). Compared with the control, the prevalence of reported runny nose was 6% less in the nutrition group (PR: 0.94, 95% CI: 0.88–1.00) (Supplemental Material). No other differences in secondary outcomes were detected across groups. Unadjusted and adjusted models yielded similar results, and comparisons between the combined WSH and WSHN groups and their respective individual intervention groups did not yield any significant differences in the respiratory outcome prevalence (Supplemental Material).

A subgroup analysis revealed that the effect of the nutrition intervention on difficulty breathing was stronger for males than females (*P*-interaction: 0.021); the prevalence of difficulty breathing in the nutrition group versus the control was 28% less for males (PR: 0.72, 95% CI: 0.56–0.94, sample size [*n*]: 760) but did not differ for females (PR: 1.14, 95% CI: 0.84–1.53, *n*: 703) (Table 3). The prevalence of ARI symptoms was 11% higher in the water versus the control group for males (PR: 1.11, 95% CI: 1.00–1.22, *n*: 724) but insignificantly different for females (PR: 0.97, 95% CI: 0.88–1.06, *n*: 812). Although treatment effects on visibly runny nose varied by child gender in the water (*P*-interaction: 0.029) and WSH (*P*-interaction: 0.027) groups compared with the control group, the prevalence ratios were statistically insignificant.

**Table 3:** Respiratory outcome prevalence ratios by child gender, interventions vs. control

	Male			Female			
Subgroup	*N*	Prevalence (%)	Prevalence Ratio (95% CI)	*N*	Prevalence (%)	Prev. Ratio (95% CI)	Interaction *P*-value
ARI*							
Control	2,254	44.8	–	2,487	47.4	–	–
Water	724	51.0	1.11 (1.00–1.22)	812	46.1	0.97 (0.88–1.06)	0.033
Sanitation	751	42.7	0.95 (0.85–1.07)	783	45.5	0.96 (0.87–1.07)	0.880
Handwashing	736	45.2	1.01 (0.91–1.12)	755	42.4	0.92 (0.83–1.01)	0.188
Nutrition	760	43.4	0.93 (0.84–1.04)	705	42.1	0.90 (0.79–1.02)	0.646
WSH	742	46.1	1.03 (0.93–1.14)	812	44.3	0.93 (0.85–1.00)	0.078
WSHN	799	43.2	0.93 (0.84–1.04)	847	47.3	1.00 (0.92–1.10)	0.316
Panting, wheezing, or difficulty breathing							
Control	2,252	13.8	–	2,485	13.0	–	–
Water	724	15.5	1.04 (0.81–1.33)	811	12.8	1.01 (0.81–1.24)	0.837
Sanitation	750	10.7	0.79 (0.61–1.02)	783	11.5	0.91 (0.73–1.14)	0.344
Handwashing	736	13.0	0.96 (0.75–1.22)	755	13.0	0.98 (0.82–1.18)	0.853
Nutrition	760	10.4	0.72 (0.56–0.94)	703	14.1	1.14 (0.84–1.53)	0.021
WSH	742	14.4	1.07 (0.84–1.37)	812	12.2	0.94 (0.74–1.18)	0.383
WSHN	799	12.4	0.86 (0.66–1.12)	847	13.1	1.03 (0.81–1.31)	0.295
Fever and ARIs							
Control	2,252	24.2	–	2,484	25.5	–	–
Water	724	28.0	1.15 (0.99–1.34)	811	26.3	1.02 (0.88–1.18)	0.257
Sanitation	751	23.3	0.97 (0.81–1.15)	783	25.5	0.99 (0.87–1.14)	0.782
Handwashing	736	25.8	1.06 (0.90–1.24)	755	21.7	0.87 (0.74–1.02)	0.088
Nutrition	760	21.6	0.85 (0.70–1.03)	703	23.0	0.91 (0.76–1.08)	0.623
WSH	742	24.9	1.05 (0.90–1.23)	812	24.1	0.92 (0.81–1.04)	0.163
WSHN	799	25.5	1.02 (0.87–1.19)	846	24.9	0.95 (0.84–1.08)	0.485
Visibly runny nose							
Control	2,110	6.7	–	2,312	6.7	–	–
Water	663	5.4	0.79 (0.54–1.18)	744	7.8	1.29 (0.97–1.72)	0.029
Sanitation	685	6.4	1.05 (0.75–1.48)	731	6.0	0.94 (0.64–1.37)	0.633
Handwashing	679	6.2	0.89 (0.66–1.22)	718	7.1	0.96 (0.70–1.33)	0.737
Nutrition	712	7.0	1.14 (0.82–1.60)	634	6.9	1.03 (0.75–1.41)	0.626
WSH	672	5.2	0.80 (0.57–1.13)	748	7.8	1.30 (0.94–1.80)	0.027
WSHN	719	5.3	0.79 (0.53–1.17)	781	6.4	0.98 (0.68–1.43)	0.343

ARI = acute respiratory infection.

* An ARI was defined as caregiver-reported cough or difficulty breathing, including panting and wheezing, within the past 7 days before the interview.

The effect of the WSH intervention on ARI symptoms was stronger during malaria season than other seasons (*P*-interaction: 0.008); the prevalence of ARI symptoms was 13% less in the WSH group than in the control group during malaria season (PR: 0.87, 95% CI: 0.77–0.97, *n*: 634) but not during the rest of the year (Table 4). The prevalence of visibly runny nose was 67% higher in the sanitation group than in the control group during malaria season (PR: 1.67, 95% CI: 1.06–2.63, *n*: 424) but not during other seasons. Although treatment effects on difficulty breathing varied by malaria seasonality in the WSH group compared with the control (*P*-interaction: 0.013), the prevalence ratios were statistically insignificant during and outside malaria season. No other effect modifications across outcomes were detected.

**Table 4:** Respiratory outcome prevalence ratios by malaria seasonality, interventions vs. control

	Malaria season			Not malaria season			
Subgroup	*N*	Prevalence (%)	Prevalence ratio (95% CI)	*N*	Prevalence (%)	Prevalence ratio (95% CI)	Interaction *P*-value
ARI*							
Control	1,693	49.3	–	3,076	44.2	–	–
Water	521	51.2	1.03 (0.93–1.16)	1,024	46.7	1.03 (0.94–1.13)	0.955
Sanitation	501	46.9	0.94 (0.83–1.07)	1,045	42.5	0.97 (0.88–1.07)	0.726
Handwashing	525	45.0	0.92 (0.83–1.03)	971	43.2	0.99 (0.89–1.10)	0.395
Nutrition	553	44.3	0.88 (0.76–1.01)	919	41.6	0.94 (0.85–1.04)	0.443
WSH	634	42.4	0.87 (0.77–0.97)	929	46.6	1.05 (0.96–1.15)	0.008
WSHN	615	45.9	0.92 (0.82–1.04)	1,042	44.6	0.99 (0.91–1.08)	0.315
Panting, wheezing, or difficulty breathing							
Control	1,691	14.7	–	3,074	12.5	–	–
Water	520	15.6	1.07 (0.77–1.48)	1,024	13.2	1.00 (0.81–1.23)	0.759
Sanitation	500	12.8	0.89 (0.71–1.11)	1,045	10.1	0.83 (0.63–1.10)	0.728
Handwashing	525	14.9	1.00 (0.80–1.26)	971	12.0	0.96 (0.78–1.18)	0.787
Nutrition	552	12.3	0.81 (0.63–1.06)	918	12.0	0.98 (0.74–1.30)	0.306
WSH	634	11.5	0.78 (0.58–1.04)	929	14.3	1.18 (0.97–1.44)	0.013
WSHN	615	14.6	0.99 (0.75–1.31)	1,042	11.5	0.91 (0.72–1.15)	0.626
Fever and ARIs							
Control	1,693	26.5	–	3,071	23.8	–	–
Water	521	29.9	1.17 (0.98–1.38)	1,023	25.5	1.04 (0.92–1.17)	0.240
Sanitation	501	25.0	0.94 (0.79–1.12)	1,045	23.9	1.01 (0.87–1.16)	0.571
Handwashing	525	24.6	0.91 (0.76–1.10)	971	23.2	0.99 (0.84–1.16)	0.570
Nutrition	553	23.7	0.84 (0.67–1.05)	917	21.3	0.90 (0.76–1.06)	0.635
WSH	634	23.2	0.88 (0.75–1.03)	929	25.2	1.05 (0.93–1.20)	0.078
WSHN	615	25.7	0.93 (0.77–1.12)	1,041	24.8	1.02 (0.89–1.16)	0.474
Visibly runny nose							
Control	1,444	6.0	–	2,978	7.1	–	–
Water	437	7.6	1.36 (0.91–2.03)	970	6.3	0.92 (0.66–1.30)	0.161
Sanitation	424	9.4	1.67 (1.06–2.63)	992	4.8	0.74 (0.53–1.04)	0.006
Handwashing	465	7.1	1.08 (0.76–1.53)	932	6.4	0.87 (0.65–1.17)	0.353
Nutrition	455	8.6	1.50 (0.94–2.38)	891	6.2	0.91 (0.67–1.24)	0.095
WSH	541	7.0	1.29 (0.80–2.10)	880	6.3	0.94 (0.68–1.29)	0.292
WSHN	508	6.1	1.10 (0.65–1.88)	992	5.7	0.80 (0.54–1.18)	0.338

ARI = acute respiratory infection.

* An ARI was defined as caregiver-reported cough or difficulty breathing, including panting and wheezing, within the past 7 days before the interview.

We detected intervention effects on ARI symptoms in the nutrition group but not in the WSHN group and examined differences in minimum dietary diversity achievement and breastfeeding rates between the two groups to better understand specific aspects of the nutritional intervention that might reduce ARI symptoms. The prevalence of achieving minimum dietary diversity was 69.0% (511/837) and 86.5% (1,007/1,222) in the nutrition group for years 1 and 2, respectively; the prevalence in the WSHN group was 62.0% and 84.3% for years 1 and 2, respectively. Compared with the nutrition-only group, the rate of achieving dietary diversity was marginally lower in the WSHN group at year 1 (PR: 0.89, 95% CI: 0.79–1.01, *P*: 0.078) and not significantly different in the WSHN group at year 2 (PR: 0.96, 95% CI: 0.92–1.01, *P*: 0.156). The prevalence of reported 6 months of exclusive breastfeeding was slightly higher in the nutrition group than in the WSHN group (*N*: 56.1% versus WSHN: 50.5%, *P*: 0.037; *C*: 32.4%), but the prevalence of any breastfeeding in the past 24 hours was not significantly different (Supplemental Material). Breastfeeding rates did not differ by child gender.

## DISCUSSION

We found limited evidence of reductions in ARI symptoms in children younger than 3 years who received LNS with counseling in infant and young child feeding and no evidence for the effect of the WSH interventions on ARIs. After 1 year of intervention, children in the nutrition arm had a lower prevalence of ARI symptoms alone (−13%) and presenting with fever (−17%), but the findings were weakened by not observing the effects on ARI symptoms after 2 years of exposure and when the nutrition intervention was combined with WASH interventions. We possibly observed reductions in ARI symptoms from the nutritional intervention only in year 1 because of more frequent exposure to community health promoters. Although the frequency of promoter visits in the month before data collection decreased in the second year, our implementation data show that most households were visited at least every other month throughout the study.^22^

Although adherence to the supplemental feeding component of the nutrition intervention, measured as the percentage of expected LNS sachets consumed, was similar in the nutrition and WSHN groups, we only found ARI symptom reductions in the nutrition group. The lack of consistency between the effects in the nutrition and WSHN groups could be due to chance. The differences could also be attributed to slightly better adherence in the nutrition arm with the dietary diversity and breastfeeding components of the intervention; it is unclear what led to the differences in compliance between the nutrition and WSHN groups. The WHO recommends that children aged 6–24 months should consume food from at least four food groups every day to ensure they are receiving micronutrients important for the development of healthy immune responses.^29^ Our findings are consistent with those of previous work in Tanzania that found low dietary diversity is a risk factor for respiratory infections.^30^ The prevalence of reported exclusive breastfeeding was higher in the nutrition and WSHN groups than in the control group. The higher breastfeeding rates in the nutrition than the WSHN group are consistent with prior evidence that breastfeeding protects against child respiratory infections.^6,30^ Our breastfeeding data are subjected to recall bias because children were 1 year old when we collected the data. We attempted to improve accuracy by correcting the data from reported exclusive breastfeeding for 6 months using reported dates of breastfeeding cessation and complementary food introduction. Breastfeeding rates in the intervention arms could also be affected by social desirability bias.^31^

Although there is strong evidence that handwashing with soap can prevent ARI, our findings emphasize that intensive behavior promotion and high adherence are necessary to achieve clinically relevant reductions in respiratory infections. In year 1, 77% of households in handwashing groups had water and soap available at a handwashing station, but only 21% had water and soap at year 2. Our assessment of adherence was limited to handwashing material availability, as we did not conduct structured observations of handwashing behavior. Our results contrast with other trials that have assessed the effects of handwashing on child respiratory health. The accompanying WASH Benefits trial in Bangladesh reported that WASH interventions reduced ARI symptoms by an average of 29% and 33% when alone and combined with nutritional interventions, respectively (Sania Ashraf, in preparation). The Bangladesh trial also reported diarrhea reductions in the sanitation, handwashing, and combined WSH arms, whereas there were no diarrhea reductions in the Kenyan trial.^22,32^ The differences between our trial and studies reporting effects of WASH interventions on respiratory health might be attributable to differences in the frequency of promoter visits and resulting adherence to interventions.^33^ Promoter visits in the WASH Benefits Bangladesh trial averaged six visits per month throughout the trial, compared with promoter visits in the Kenyan trial averaging one visit every 1–2 months.^33^ We may not have observed the effects in the individual and combined WSH groups because of low adherence among participants, but levels of adherence were comparable to—or better than—what a government or nongovernmental organization might achieve at scale.^22^

Peaks in ARI and pneumonia cases have been observed during malaria seasons in rural Kenya.^27^ Defining the malaria seasons as January–February and June–August, our data showed a slight increase in ARI cases in the control group during January and February in year 1 and peaks in ARI cases in January–February and June–August in year 2 (Supplemental Material). Subgroup analyses revealed that combined WSH interventions reduced the prevalence of ARI symptoms during malaria seasons but not during other months. Similar environmental conditions might favor malaria and respiratory pathogen transmission, or the correlation between malaria and ARI could be explained by an overlap of symptoms. Associations between malaria and bacteremia have been found in Kenyan children, but direct associations between malaria and respiratory illness have not been established.^34^ Males might be more susceptible to ARIs than females, and mothers might be more likely to exclusively breastfeed males than females.^3,35^ We observed a lower prevalence of difficulty breathing in males (compared with females) in the nutrition group versus the control group. Our data did not detect differences in rates of exclusive breastfeeding for 6 months between males and females, but it is subjected to recall and social desirability bias, as described earlier.

The main limitations of this study include low adherence to some of the targeted behaviors, reliance on caregiver-reported outcomes (excluding visibly runny nose) subjected to bias, and lack of data available to assess intervention effects on the WHO-defined pneumonia. The indicators of respiratory illness in this analysis are nonspecific and likely attributable to mild infections that are unlikely to lead to child death. Our data are unable to identify severe, life-threatening, lower respiratory tract infections. However, upper respiratory tract infections in children and their family members can often make children more susceptible to pneumonia.^36^

Our findings suggest provision of dual tippy taps coupled with behavior change promotion will not reduce ARI symptoms among children in rural Kenya. Likewise, coupling behavior change promotion and provision of chlorine for water treatment, feces management hardware, or latrine slabs should not be expected to reduce respiratory infections. Poor uptake, especially in year 2, is a possible explanation for the lack of effects of handwashing and water quality interventions on ARI symptoms. Because reported consumption of LNS was high in the nutrition and WSHN groups, one explanation for the mixed impacts on ARIs between the two groups is that the counseling components of the nutritional intervention (e.g., promotion of minimum dietary diversity requirements and exclusive breastfeeding for 6 months) may have contributed to ARI reductions in the individual nutrition group. Although more intensive promotion and higher adherence may have yielded positive effects of handwashing and nutritional interventions on ARI symptoms, the results from this trial are relevant for large-scale programs that have limited resources for promoter–participant interactions.

## Supplemental material

Supplemental materials
