# Supplementary material for: Effects of Individual and Combined Water, Sanitation, Handwashing, and Nutritional Interventions on Child Respiratory Infections in Rural Kenya: A Cluster-Randomized Controlled Trial
Source: Am J Trop Med Hyg. 2020 Mar 30;102(6):1286–95. doi: 10.4269/ajtmh.19-0779 (PMC7253138; doi:10.4269/ajtmh.19-0779)

Supplementary Information for
WASH Benefits Kenya Respiratory Infections Analysis

TABLE OF CONTENTS

CONSORT Abstract Checklist 1

CONSORT Checklist 2

Table S1: Baseline characteristics of participating households and households lost to follow-up 5

Table S2: Indicators of intervention adoption one year after intervention delivery 6

Table S3: Indicators of intervention adoption two years after intervention delivery 7

Table S4: Unadjusted effects of interventions on prevalence of respiratory outcomes, interventions vs. control 8

Table S5: Adjusted effects of interventions on prevalence of respiratory outcomes, interventions vs. control, years one and two combined 9

Table S6: Effects of interventions on prevalence of respiratory outcomes, combined (intervention) vs. single (reference) intervention arms, years one and two combined 10

Table S7: Unadjusted effects of interventions on prevalence of respiratory outcomes, combined (intervention) vs. single (reference) intervention arms, year one only 11

Table S8: Respiratory outcome prevalence ratios by index child status, interventions vs. control 12

Table S9: Respiratory outcome prevalence differences by index child status, interventions vs. control 13

Table S10: Respiratory outcome prevalence differences by child sex, interventions vs. control 14

Table S11: Respiratory outcome prevalence differences by malaria seasonality, interventions vs. control 15

Table S12: Prevalence of breastfeeding indicators 16

Table S13: Prevalence of breastfeeding indicators, stratified by child sex 16

Table S14: Unadjusted effects of interventions on prevalence of reported runny nose, intervention vs. control 17

Table S15: Reported runny nose prevalence ratios and differences by malaria seasonality interventions vs. control 17

Figure S1: Acute respiratory illness by calendar month 18

CONSORT Abstract Checklist

Item	Standard Description	Description for Cluster Trials	Reported in Line:
Title	Identification of the study as randomised	Identification of the study as cluster-randomised	2
Trial design	Description of the trial design (e.g. parallel, cluster, non-inferiority)	Definition of cluster and description of how the design features apply to the clusters	31
Methods			
Participants	Eligibility criteria for participants and the settings where the data were collected	Eligibility criteria for clusters	32
Interventions	Interventions intended for each group	Whether interventions pertain to the cluster level, the individual participant level or both	33-37
Objective	Specific objective or hypothesis		30-31
Outcome	Clearly defined primary outcome for this report	Whether outcome measures pertain to the cluster level, the individual participant level or both	38-39
Randomization	How participants were allocated to interventions		33-34
Blinding (masking)	Whether or not participants, care givers, and those assessing the outcomes were blinded to group assignment		39
Results			
Numbers randomized	Number of participants randomized to each group	Number of clusters randomized to each group	40
Recruitment	Trial status		Not applicable – publication indicates this is final report
Numbers analysed	Number of participants analysed in each group	For each group, number of clusters included in each analysis	41
Outcome	For the primary outcome, a result for each group and the estimated effect size and its precision	Results at the individual or cluster level as applicable and a coefficient of intracluster correlation (ICC or k) for each primary outcome	42
Harms	Important adverse events or side effects		Not applicable
Conclusions	General interpretation of the results		42-45
Trial registration	Registration number and name of trial register		45
Funding	Source of funding		45

CONSORT Checklist

Section/Topic	Item No	Standard Checklist item	Extension for cluster designs	Extension for multi-arm trials	Reported in Section:
**Title and abstract**					
	1a	Identification as a randomised trial in the title	Identification as a cluster randomised trial in the title	Identification as a multi-arm randomised trial in the title or an indication of the number of treatment groups that the participants were randomly assigned to	Title
	1b	Structured summary of trial design, methods, results, and conclusions (for specific guidance see CONSORT for abstracts)[i, ii]	See table 2	Specification of the number of treatment groups; details of any groups added or dropped	Abstract
**Introduction**					
**Background and objectives**	2a	Scientific background and explanation of rationale	Rationale for using a cluster design	Rationale for using a multi-arm design	Introduction
	2b	Specific objectives or hypotheses	Whether objectives pertain to the cluster level, the individual participant level or both	Specification of the research question referring to all of the treatment groups<br>Clear statement of all hypotheses to be tested and the primary comparisons involved	Introduction, last paragraph
**Methods**					
**Trial design**	3a	Description of trial design (such as parallel, factorial) including allocation ratio	Definition of cluster and description of how the design features apply to the clusters	Specification of the number of treatment groups	Materials and Methods (Study design)
	3b	Important changes to methods after trial commencement (such as eligibility criteria), with reasons		Details of any treatment groups added or dropped (if relevant), with reasons, and/or changes to the allocation ratio	Not applicable
**Participants**	4a	Eligibility criteria for participants	Eligibility criteria for clusters		Materials and Methods (Study design)
	4b	Settings and locations where the data were collected			Materials and Methods (Study design)
**Interventions**	5	The interventions for each group with sufficient details to allow replication, including how and when they were actually administered	Whether interventions pertain to the cluster level, the individual participant level or both		Materials and Methods (Procedures)
**Outcomes**	6a	Completely defined pre-specified primary and secondary outcome measures, including how and when they were assessed	Whether outcome measures pertain to the cluster level, the individual participant level or both		Materials and Methods (Outcomes)
	6b	Any changes to trial outcomes after the trial commenced, with reasons			Not applicable
**Sample size**	7a	How sample size was determined	Method of calculation, number of clusters(s) (and whether equal or unequal cluster sizes are assumed), cluster size, a coefficient of intracluster correlation (ICC or *k*), and an indication of its uncertainty	Planned sample size with details of how it was determined for each primary comparison	Materials and Methods (Statistical analysis)
	7b	When applicable, explanation of any interim analyses and stopping guidelines			Not applicable
**Randomisation:**					
**Sequence generation**	8a	Method used to generate the random allocation sequence			Materials and Methods (Study design)
	8b	Type of randomisation; details of any restriction (such as blocking and block size)	Details of stratification or matching if used		Materials and Methods (Study design)

**Allocation concealment mechanism**	9	Mechanism used to implement the random allocation sequence (such as sequentially numbered containers), describing any steps taken to conceal the sequence until interventions were assigned	Specification that allocation was based on clusters rather than individuals and whether allocation concealment (if any) was at the cluster level, the individual participant level or both		Materials and Methods (Study design)
**Implementation**	10	Who generated the random allocation sequence, who enrolled participants, and who assigned participants to interventions	Replace by 10a, 10b and 10c		
	10a		Who generated the random allocation sequence, who enrolled clusters, and who assigned clusters to interventions		Materials and Methods (Study design)
	10b		Mechanism by which individual participants were included in clusters for the purposes of the trial (such as complete enumeration, random sampling)		Materials and Methods (Study design)
	10c		From whom consent was sought (representatives of the cluster, or individual cluster members, or both), and whether consent was sought before or after randomisation		Materials and Methods (Study design)
**Blinding**	11a	If done, who was blinded after assignment to interventions (for example, participants, care providers, those assessing outcomes) and how			Materials and Methods (Study design)
	11b	If relevant, description of the similarity of interventions			Not applicable
**Statistical methods**	12a	Statistical methods used to compare groups for primary and secondary outcomes	How clustering was taken into account	Explicitly state if no adjustments for multiplicity were applied; if adjustments were applied, state the method used	Materials and Methods (Statistical analysis, second paragraph)
	12b	Methods for additional analyses, such as subgroup analyses and adjusted analyses			Materials and Methods (Statistical analysis)
**Results**					
**Participant flow (a diagram is strongly recommended)**	13a	For each group, the numbers of participants who were randomly assigned, received intended treatment, and were analysed for the primary outcome	For each group, the numbers of clusters that were randomly assigned, received intended treatment, and were analysed for the primary outcome		Results; Figure 1
	13b	For each group, losses and exclusions after randomisation, together with reasons	For each group, losses and exclusions for both clusters and individual cluster members		Results; Figure 1
**Recruitment**	14a	Dates defining the periods of recruitment and follow-up		If periods of recruitment and follow-up are different across treatment groups (eg, groups were added or dropped), the periods of recruitment and follow-up, reason(s) for the differences, and any statistical implications should be described	Results, first paragraph
	14b	Why the trial ended or was stopped			Not applicable
**Baseline data**	15	A table showing baseline demographic and clinical characteristics for each group	Baseline characteristics for the individual and cluster levels as applicable for each group		Results; Table 1

**Numbers analysed**	16	For each group, number of participants (denominator) included in each analysis and whether the analysis was by original assigned groups	For each group, number of clusters included in each analysis		Results; Figure 1
**Outcomes and estimation**	17a	For each primary and secondary outcome, results for each group, and the estimated effect size and its precision (such as 95% confidence interval)	Results at the individual or cluster level as applicable and a coefficient of intracluster correlation (ICC or k) for each primary outcome	Results for each prespecified comparison of treatment groups	Results; Figure 2; Figure 3; Table 2
	17b	For binary outcomes, presentation of both absolute and relative effect sizes is recommended			Results; Figure 2; Figure 3; Table 2; Table S3
**Ancillary analyses**	18	Results of any other analyses performed, including subgroup analyses and adjusted analyses, distinguishing pre-specified from exploratory			Results; Table 3; Table 4; Table S4; Table S5; Table S6; Table S7; Table S11; Table S12; Table S13; Table S14; Table S15
**Harms**	19	All important harms or unintended effects in each group (for specific guidance see CONSORT for harms[iii])			Not applicable
**Discussion**					
**Limitations**	20	Trial limitations, addressing sources of potential bias, imprecision, and, if relevant, multiplicity of analyses			Discussion, second to last paragraph
**Generalisability**	21	Generalisability (external validity, applicability) of the trial findings	Generalisability to clusters and/or individual participants (as relevant)		Discussion, last paragraph
**Interpretation**	22	Interpretation consistent with results, balancing benefits and harms, and considering other relevant evidence			Discussion
**Other information**					
**Registration**	23	Registration number and name of trial registry			Materials and Methods (Study design)
**Protocol**	24	Where the full trial protocol can be accessed, if available			Reference 21<br>Methods (Study design, first paragraph)
**Funding**	25	Sources of funding and other support (such as supply of drugs), role of funders			Financial support

[i] Hopewell S, Clarke M, Moher D, Wager E, Middleton P, Altman DG, et al. CONSORT for reporting randomised trials in journal and conference abstracts. *Lancet* 2008, 371:281-283

[ii] Hopewell S, Clarke M, Moher D, Wager E, Middleton P, Altman DG at al (2008) CONSORT for reporting randomized controlled trials in journal and conference abstracts: explanation and elaboration. *PLoS Med* 5(1): e20

[iii] Ioannidis JP, Evans SJ, Gotzsche PC, O'Neill RT, Altman DG, Schulz K, Moher D. Better reporting of harms in randomized trials: an extension of the CONSORT statement. *Ann Intern Med* 2004; 141(10):781-788.

Table S1: Baseline characteristics of participating households and households lost to follow-up

No. of households:	Households in Analysis (N=7057)	Households Lost to Follow-Up (N=1189)
	n (%)/mean (SD)	n (%)/mean (SD)
**Maternal**		
Age (years)	26 (6)	25 (7)
Completed primary school	3345 (47%)	568 (48%)
**Paternal**		
Completed primary school	4060 (62%)	611 (59%)
Works in agriculture	2909 (43%)	408 (38%)
**Household**		
Number of persons	5 (2)	5 (3)
Has electricity	499 (7%)	63 (5%)
Has a cement floor	401 (6%)	73 (6%)
**Drinking water**		
Has protected primary drinking water source*	5248 (75%)	883 (75%)
Stored water observed at home	5760 (82%)	919 (78%)
Reported treating currently stored water	728 (13%)	113 (12%)
**Sanitation**		
Daily defecating in the open		
Children: 3-<8 years	587 (13%)	100 (15%)
Children: 0-<3 years	3006 (78%)	410 (76%)
Latrine		
Owned by compound	5831 (83%)	951 (80%)
Household has access to a latrine with slab or ventilation pipe	1116 (17%)	199 (18%)
Visible stool on slab or floor	3160 (49%)	529 (49%)
Has a potty	157 (2%)	33 (3%)
Human feces observed in the compound	604 (9%)	93 (8%)
**Handwashing**		
Handwashing station has water and soap	413 (6%)	65 (5%)
**Nutrition**		
Moderate-to-severe household hunger†	736 (10%)	172 (15%)

Percentages were calculated from smaller denominators than those shown at the top of the table for all variables because of missing values.
* Protected water sources include borewells, protected springs, protected dug wells, rainwater collection, and piped water into the home or yard/plot
† Assessed by Household Hunger Scale

Table S2: Indicators of intervention adoption one year after intervention delivery

No. of households:	Control (N=1015)	Water Treatment (N=468)	Sanitation (N=464)	Handwashing (N=483)	Water + Sanitation + Handwashing (N=522)	Nutrition (N=446)	Nutrition + Water + Sanitation + Handwashing (N=485)
	n (%)	n (%)	n (%)	n (%)	n (%)	n (%)	n (%)
**Intervention Promotion**							
Visited by a promoter in the past month	655 (69%)	334 (76%)	328 (75%)	329 (71%)	378 (75%)	341 (80%)	384 (82%)
**Water Storage**							
Had stored drinking water available in household	899 (90%)	402 (86%)	401 (88%)	439 (91%)	424 (82%)	415 (94%)	381 (79%)
Obtained stored water from protected water source*	671 (80%)	283 (76%)	297 (82%)	314 (76%)	300 (75%)	313 (81%)	276 (78%)
**Water Treatment**							
Reported treating currently stored water	92 (10%)	213 (53%)	48 (12%)	49 (11%)	251 (59%)	36 (9%)	208 (55%)
Used Vestergaard LifeStraw® family filter	19 (2%)	3 (1%)	6 (1%)	5 (1%)	1 (0%)	5 (1%)	1 (0%)
Boiled water	12 (1%)	2 (0%)	4 (1%)	4 (1%)	0 (0%)	3 (1%)	1 (0%)
Treated with chlorine	5 (6%)	206 (51%)	29 (7%)	35 (8%)	250 (59%)	22 (5%)	205 (54%)
Detectable total chlorine in stored drinking water	24 (3%)	155 (40%)	23 (6%)	19 (5%)	187 (44%)	9 (2%)	157 (43%)
**Sanitation**							
Household has access to a latrine with slab or ventilation pipe	170 (18%)	73 (16%)	402 (90%)	64 (14%)	458 (89%)	63 (15%)	417 (89%)
Has a potty	22 (2%)	7 (2%)	446 (97%)	21 (4%)	507 (98%)	5 (1%)	461 (96%)
Child feces safely disposed†	331 (37%)	153 (37%)	313 (77%)	153 (36%)	320 (70%)	154 (40%)	285 (67%)
**Handwashing**							
Handwashing station has water and soap	120 (12%)	53 (11%)	49 (11%)	372 (77%)	407 (78%)	61 (14%)	377 (78%)
**Nutrition**							
LNS sachets consumed (% of expected)	-	-	-	-	-	4984/5264 (95%)	5279/5530 (95%)

Percentages were calculated from smaller denominators than those shown at the top of the table for all variables because of missing values.

* Protected water sources include borewells, protected springs, protected dug wells, rainwater collection, and piped water into the home or yard/plot

† Safe child faeces disposal was defined as the index child's last defecation in a latrine, or diaper or potty and rinsed into a latrine or buried

Table S3: Indicators of intervention adoption two years after intervention delivery

No. of households:	Control (N=1506)	Water Treatment (N=691)	Sanitation (N=713)	Handwashing (N=684)	Water + Sanitation + Handwashing (N=676)	Nutrition (N=652)	Nutrition + Water + Sanitation + Handwashing (N=734)
	n (%)	n (%)	n (%)	n (%)	n (%)	n (%)	n (%)
**Intervention Promotion**							
Visited by a promoter in the past month	493 (34%)	252 (37%)	275 (40%)	229 (34%)	241 (37%)	252 (40%)	257 (37%)
**Water Storage**							
Had stored drinking water available in household	1406 (94%)	631 (92%)	639 (91%)	640 (94%)	597 (89%)	613 (95%)	647 (89%)
Obtained stored water from protected water source*	1196 (85%)	498 (79%)	554 (87%)	551 (86%)	480 (80%)	497 (81%)	525 (81%)
**Water Treatment**							
Reported treating currently stored water	217 (15%)	226 (36%)	93 (15%)	81 (13%)	196 (33%)	74 (12%)	202 (31%)
Used Vestergaard LifeStraw® family filter	28 (2%)	8 (1%)	12 (2%)	4 (1%)	0 (0%)	3 (0%)	6 (1%)
Boiled water	22 (2%)	4 (1%)	8 (1%)	11 (2%)	8 (1%)	9 (1%)	6 (1%)
Treated with chlorine	121 (9%)	208 (33%)	53 (8%)	47 (7%)	184 (31%)	47 (8%)	190 (29%)
Detectable total chlorine in stored drinking water	48 (3%)	150 (24%)	19 (3%)	18 (3%)	120 (20%)	17 (3%)	127 (20%)
**Sanitation**							
Household has access to a latrine with slab or ventilation pipe	287 (20%)	126 (19%)	530 (78%)	120 (19%)	530 (82%)	98 (16%)	558 (80%)
Has a potty	76 (5%)	30 (4%)	607 (86%)	34 (5%)	566 (85%)	31 (5%)	609 (84%)
Child feces safely disposed†	149 (11%)	52 (8%)	239 (37%)	62 (10%)	204 (34%)	49 (8%)	217 (33%)
**Handwashing**							
Handwashing station has water and soap	131 (9%)	51 (7%)	58 (8%)	159 (23%)	132 (20%)	76 (12%)	154 (21%)
**Nutrition**							
LNS sachets consumed (% of expected)	-	-	-	-	-	3566/3122 (114%)	4046/3486 (116%)

Percentages were calculated from smaller denominators than those shown at the top of the table for all variables because of missing values.

* Protected water sources include borewells, protected springs, protected dug wells, rainwater collection, and piped water into the home or yard/plot

† Safe child faeces disposal was defined as the index child's last defecation in a latrine, or diaper or potty and rinsed into a latrine or buried

Table S4: Unadjusted effects of interventions on prevalence of respiratory outcomes, interventions vs. control

	Years One and Two Combined				Year One Only			
**Outcome, Arm**	**N**	**Prev.**	**Prev. Difference (95% CI)**	**P-value**	**N**	**Prev.**	**Prev. Difference (95% CI)**	**P-value**
**Acute respiratory infection (ARI)***								
Control	4769	46.0%			2347	48.1%		
Water	1545	48.2%	0.02 (-0.02 - 0.05)	0.397	764	50.0%	0.01 (-0.04 - 0.06)	0.704
Sanitation	1546	43.9%	-0.02 (-0.05 - 0.02)	0.285	755	45.6%	-0.02 (-0.06 - 0.02)	0.402
Handwashing	1496	43.8%	-0.02 (-0.05 - 0.02)	0.345	738	47.6%	0.01 (-0.04 - 0.05)	0.769
Nutrition	1472	42.6%	-0.04 (-0.08 - 0.00)	0.050	735	41.8%	-0.06 (-0.12 - -0.01)	0.024
WSH	1563	44.9%	-0.01 (-0.04 - 0.02)	0.470	788	47.2%	-0.01 (-0.05 - 0.04)	0.671
WSHN	1657	45.1%	-0.01 (-0.05 - 0.02)	0.360	833	46.5%	-0.02 (-0.07 - 0.02)	0.275
**Panting, wheezing or difficulty breathing**								
Control	4765	13.3%			2344	11.5%		
Water	1544	14.0%	0.00 (-0.02 - 0.03)	0.788	764	12.8%	0.01 (-0.02 -0.05)	0.526
Sanitation	1545	11.0%	-0.02 (-0.04 - 0.00)	0.074	754	10.5%	-0.01 (-0.04 - 0.02)	0.591
Handwashing	1496	13.0%	0.00 (-0.02 - 0.02)	0.785	738	13.1%	0.01 (-0.02 - 0.05)	0.389
Nutrition	1470	12.1%	-0.01 (-0.04 - 0.01)	0.400	734	10.4%	-0.01 (-0.04 - 0.02)	0.598
WSH	1563	13.2%	0.00 (-0.02 - 0.02)	0.976	788	13.5%	0.02 (-0.02 - 0.06)	0.378
WSHN	1657	12.7%	-0.01 (-0.03 - 0.02)	0.541	833	10.3%	-0.01 (-0.04 - 0.02)	0.462
**Fever and ARI**								
Control	4764	24.7%			2345	26.1%		
Water	1544	27.0%	0.02 (-0.01 - 0.05)	0.134	763	27.3%	0.01 (-0.03 - 0.05)	0.625
Sanitation	1546	24.3%	0.00 (-0.03 - 0.02)	0.716	755	24.9%	-0.02 (-0.05 - 0.02)	0.416
Handwashing	1496	23.7%	-0.01 (-0.04 - 0.02)	0.505	738	25.9%	0.00 (-0.04 - 0.03)	0.937
Nutrition	1470	22.2%	-0.03 (-0.06 - 0.00)	0.043	734	21.8%	-0.05 (-0.09 - 0.00)	0.033
WSH	1563	24.4%	-0.01 (-0.03 - 0.02)	0.675	788	25.5%	-0.01 (-0.04 - 0.02)	0.589
WSHN	1656	25.1%	0.00 (-0.03 - 0.02)	0.776	832	25.0%	-0.02 (-0.05 - 0.02)	0.365
**Visibly runny nose**								
Control	4422	6.7%			2035	6.9%		
Water	1407	6.7%	0.00 (-0.01 - 0.02)	0.749	647	7.3%	0.01 (-0.02 - 0.04)	0.576
Sanitation	1416	6.2%	0.00 (-0.02 - 0.02)	0.952	632	4.3%	-0.03 (-0.05 - 0.00)	0.050
Handwashing	1397	6.7%	0.00 (-0.02 - 0.01)	0.535	650	7.8%	0.01 (-0.02 - 0.04)	0.654
Nutrition	1346	7.0%	0.01 (-0.01 - 0.02)	0.522	618	7.6%	0.01 (-0.02 - 0.04)	0.468
WSH	1421	6.5%	0.00 (-0.01 - 0.02)	0.698	662	6.8%	0.00 (-0.03 - 0.03)	0.889
WSHN	1500	5.9%	-0.01 (-0.03 - 0.01)	0.418	687	4.8%	-0.02 (-.05 - 0.00)	0.052

*An ARI was defined as caregiver-reported cough or difficulty breathing, including panting and wheezing, within the past seven days prior to the interview

Table S5: Adjusted effects of interventions on prevalence of respiratory outcomes, interventions vs. control, years one and two combined

Outcome, Arm	N	Prev.	Prev. Ratio (95% CI)	P-value	Prev. Difference (95% CI)	P-value
**Acute respiratory infection (ARI)***						
Control	4769	46.0%				
Water	1545	48.2%	1.01 (0.94 - 1.09)	0.736	0.01 (-0.03 - 0.04)	0.727
Sanitation	1546	43.9%	0.97 (0.90 - 1.05)	0.512	-0.01 (-0.05 - 0.02)	0.514
Handwashing	1496	43.8%	0.95 (0.89 - 1.03)	0.210	-0.02 (-0.05 - 0.01)	0.233
Nutrition	1472	42.6%	0.92 (0.85 - 1.00)	0.060	-0.03 (-0.07 - 0.00)	0.058
WSH	1563	44.9%	0.96 (0.90 - 1.03)	0.216	-0.02 (-0.05 - 0.01)	0.209
WSHN	1657	45.1%	1.00 (0.93 - 1.07)	0.911	0.00 (-0.03 - 0.03)	0.890
**Panting, wheezing or difficulty breathing**						
Control	4765	13.3%				
Water	1544	14.0%	0.99 (0.84 - 1.17)	0.928	0.00 (-0.03 - 0.02)	0.847
Sanitation	1545	11.0%	0.93 (0.79 - 1.09)	0.371	-0.01 (-0.03 - 0.01)	0.294
Handwashing	1496	13.0%	0.97 (0.86 - 1.10)	0.680	0.00 (-0.02 - 0.01)	0.726
Nutrition	1470	12.1%	0.93 (0.79 - 1.10)	0.403	-0.01 (-0.03 - 0.01)	0.313
WSH	1563	13.2%	0.96 (0.82 - 1.11)	0.563	0.00 (-0.02 - 0.01)	0.603
WSHN	1657	12.7%	1.02 (0.87 - 1.20)	0.800	0.00 (-0.02 - 0.02)	0.878
**Fever and ARI**						
Control	4764	24.7%				
Water	1544	27.0%	1.06 (0.95 - 1.18)	0.328	0.01 (-0.02 - 0.04)	0.329
Sanitation	1546	24.3%	1.00 (0.89 - 1.12)	0.997	0.00 (-0.03 - 0.03)	0.914
Handwashing	1496	23.7%	0.94 (0.82 - 1.06)	0.285	-0.02 (-0.04 - 0.01)	0.277
Nutrition	1470	22.2%	0.88 (0.77 - 1.00)	0.048	-0.03 (-0.06 - 0.00)	0.052
WSH	1563	24.4%	0.98 (0.89 - 1.08)	0.676	0.00 (-0.03 - 0.02)	0.759
WSHN	1656	25.1%	1.01 (0.92 - 1.11)	0.844	0.00 (-0.02 - 0.03)	0.820
**Visibly runny nose**						
Control	4422	6.7%				
Water	1407	6.7%	0.96 (0.72 - 1.28)	0.770	0.00 (-0.02 - 0.02)	0.949
Sanitation	1416	6.2%	0.96 (0.72 - 1.29)	0.783	0.00 (-0.02 - 0.02)	0.965
Handwashing	1397	6.7%	0.83 (0.64 - 1.09)	0.176	-0.01 (-0.03 - 0.01)	0.170
Nutrition	1346	7.0%	1.07 (0.82 - 1.41)	0.609	0.01 (-0.01 - 0.02)	0.577
WSH	1421	6.5%	1.19 (0.91 - 1.56)	0.211	0.01 (-0.01 - 0.03)	0.338
WSHN	1500	5.9%	0.90 (0.64 - 1.27)	0.551	-0.0 (-0.03 - 0.01)	0.570

*An ARI was defined as caregiver-reported cough or difficulty breathing, including panting and wheezing, within the past seven days prior to the interview

Table S6: Effects of interventions on prevalence of respiratory outcomes, combined (intervention) vs. single (reference) intervention arms, years one and two combined

	Unadjusted				Adjusted			
**Outcome, Arm**	**Prev. Ratio (95% CI)**	**P-value**	**Prev. Difference (95% CI)**	**P-value**	**Prev. Ratio (95% CI)**	**P-value**	**Prev. Difference (95% CI)**	**P-value**
**Acute respiratory infection (ARI)***								
WSH vs. Water	0.94 (0.86 - 1.03)	0.217	-0.03 (-0.07 - 0.02)	0.218	0.94 (0.86 - 1.03)	0.185	-0.03 (-0.07 - 0.01)	0.164
WSH vs. Sanitation	1.04 (0.94 - 1.15)	0.461	0.02 (-0.03 - 0.06)	0.461	1.01 (0.92 - 1.11)	0.823	0.00 (-0.04 - 0.04)	0.880
WSH vs. Handwashing	1.03 (0.94 - 1.13)	0.496	0.01 (-0.03 - 0.05)	0.495	1.04 (0.95 - 1.14)	0.369	0.02 (-0.02 - 0.06)	0.384
WSHN vs. WSH	1.01 (0.92 - 1.11)	0.834	0.00 (-0.04 - 0.05)	0.834	1.08 (0.99 - 1.18)	0.090	0.03 (-0.01 - 0.07)	0.115
WSHN vs. Nutrition	1.07 (0.98 - 1.16)	0.133	0.03 (-0.01 - 0.07)	0.130	1.10 (1.02 - 1.19)	0.015	0.04 (0.01 - 0.08)	0.021
**Panting, wheezing or difficulty breathing**								
WSH vs. Water	0.98 (0.79 - 1.20)	0.827	0.00 (-0.03 - 0.02)	0.828	0.95 (0.76 - 1.17)	0.620	-0.01 (-0.04 - 0.02)	0.567
WSH vs. Sanitation	1.24 (1.00 - 1.55)	0.054	0.03 (0.00 - 0.05)	0.052	1.24 (0.99 - 1.55)	0.061	0.02 (0.00 - 0.04)	0.108
WSH vs. Handwashing	1.07 (0.85 - 1.35)	0.557	0.01 (-0.02 - 0.04)	0.557	1.04 (0.84 - 1.29)	0.724	0.00 (-0.02 - 0.03)	0.721
WSHN vs. WSH	0.97 (0.78 - 1.20)	0.773	0.00 (-0.03 - 0.02)	0.772	1.02 (0.82 - 1.27)	0.843	0.01 (-0.02 - 0.03)	0.608
WSHN vs. Nutrition	0.97 (0.77 - 1.22)	0.796	0.00 (-0.03 - 0.03)	0.796	1.10 (0.90 - 1.33)	0.360	0.01 (-0.02 - 0.03)	0.618
**Fever and ARI**								
WSH vs. Water	0.93 (0.81 - 1.06)	0.280	-0.02 (-0.05 - 0.02)	0.283	0.94 (0.83 - 1.07)	0.336	-0.02 (-0.05 - 0.02)	0.302
WSH vs. Sanitation	1.04 (0.90 - 1.21)	0.585	0.01 (-0.03 - 0.05)	0.585	0.99 (0.87 - 1.13)	0.905	0.00 (-0.03 - 0.03)	0.996
WSH vs. Handwashing	1.07 (0.92 - 1.25)	0.351	0.02 (-0.02 - 0.05)	0.348	1.12 (0.97 - 1.30)	0.134	0.02 (-0.01 - 0.06)	0.186
WSHN vs. WSH	1.01 (0.88 - 1.16)	0.841	0.00 (-0.03 - 0.04)	0.841	1.06 (0.93 - 1.20)	0.400	0.01 (-0.02 - 0.04)	0.404
WSHN vs. Nutrition	1.15 (1.00 -1.33)	0.046	0.03 (0.00 - 0.07)	0.044	1.20 (1.04 - 1.39)	0.012	0.04 (0.01 - 0.08)	0.014
**Visibly runny nose**								
WSH vs. Water	0.98 (0.70 - 1.36)	0.901	0.00 (-0.02 - 0.02)	0.901	1.19 (0.85 - 1.66)	0.324	0.01 (-0.01 - 0.04)	0.334
WSH vs. Sanitation	1.20 (0.84 - 1.71)	0.318	0.01 (-0.01 - 0.03)	0.314	1.40 (0.93 - 2.13)	0.110	0.01 (-0.01 - 0.04)	0.200
WSH vs. Handwashing	1.11 (0.81 - 1.53)	0.504	0.01 (-0.01 - 0.03)	0.502	1.36 (0.97 - 1.90)	0.073	0.02 (0.00 - 0.04)	0.119
WSHN vs. WSH	0.80 (0.54 - 1.20)	0.282	-0.01 (-0.04 - 0.01)	0.269	0.83 (0.55 - 1.24)	0.361	-0.02 (-0.04 - 0.01)	0.235
WSHN vs. Nutrition	0.82 (0.56 - 1.19)	0.291	-0.01 (-0.04 - 0.01)	0.275	0.78 (0.49 - 1.25)	0.305	-0.01 (-0.04 - 0.01)	0.302

*An ARI was defined as caregiver-reported cough or difficulty breathing, including panting and wheezing, within the past seven days prior to the interview

Table S7: Unadjusted effects of interventions on prevalence of respiratory outcomes, combined (intervention) vs. single (reference) intervention arms, year one only

Outcome, Arm	Prev. Ratio (95% CI)	P-value	Prev. Difference (95% CI)	P-value
**Acute respiratory infection (ARI)***				
WSH vs. Water	0.98 (0.87 - 1.11)	0.751	-0.01 (-0.07 - 0.05)	0.751
WSH vs. Sanitation	1.07 (0.94 - 1.21)	0.317	0.03 (-0.03 - 0.09)	0.318
WSH vs. Handwashing	1.00 (0.88 - 1.13)	0.943	0.00 (-0.06 - 0.06)	0.943
WSHN vs. WSH	0.98 (0.86 - 1.12)	0.788	-0.01 (-0.07 - 0.05)	0.788
WSHN vs. Nutrition	1.13 (0.99 - 1.28)	0.061	0.05 (0.00 - 0.11)	0.058
**Panting, wheezing or difficulty breathing**				
WSH vs. Water	1.08 (0.75 - 1.55)	0.685	0.01 (-0.04 - 0.06)	0.685
WSH vs. Sanitation	1.33 (0.92 - 1.93)	0.124	0.03 (-0.01 - 0.08)	0.119
WSH vs. Handwashing	1.09 (0.78 - 1.53)	0.622	0.01 (-0.03 - 0.06)	0.625
WSHN vs. WSH	0.77 (0.56 - 1.05)	0.100	-0.03 (-0.07 - 0.01)	0.106
WSHN vs. Nutrition	0.87 (0.62 - 1.22)	0.426	-0.01 (-0.05 - 0.02)	0.424
**Fever and ARI**				
WSH vs. Water	0.96 (0.80 - 1.16)	0.702	-0.01 (-0.06 - 0.04)	0.702
WSH vs. Sanitation	1.11 (0.81 - 1.35)	0.325	0.02 (-0.02 - 0.07)	0.323
WSH vs. Handwashing	1.01 (0.84 - 1.20)	0.956	0.00 (-0.04 - 0.05)	0.956
WSHN vs. WSH	0.96 (0.80 - 1.16)	0.683	-0.01 (-0.06 - 0.04)	0.683
WSHN vs. Nutrition	1.17 (0.94 - 1.46)	0.166	0.04 (-0.01 - 0.09)	0.163
**Visibly runny nose**				
WSH vs. Water	0.98 (0.62 - 1.57)	0.944	0.00 (-0.04 - 0.03)	0.944
WSH vs. Sanitation	1.77 (0.97 - 3.22)	0.064	0.03 (0.00 - 0.06)	0.06
WSH vs. Handwashing	0.94 (0.58 - 1.52)	0.803	0.00 (-0.04 - 0.03)	0.803
WSHN vs. WSH	0.62 (0.38 - 1.02)	0.060	-0.03 (-0.06 - 0.00)	0.071
WSHN vs. Nutrition	0.59 (0.36 - 0.96)	0.033	-0.03 (-0.06 - 0.00)	0.042

*An ARI was defined as caregiver-reported cough or difficulty breathing, including panting and wheezing, within the past seven days prior to the interview

Table S8: Respiratory outcome prevalence ratios by index child status, interventions vs. control

Subgroup	Index Children			Non-index Children			
	N	Prev.	Prev. Ratio (95% CI)	N	Prev.	Prev. Ratio (95% CI)	Interaction P-value
**Acute respiratory infection (ARI)***							
Control	4331	46.5%		438	41.1%		
Water	1395	48.7%	1.03 (0.96 - 1.11)	150	44.0%	1.04 (0.81 - 1.33)	0.965
Sanitation	1383	44.9%	0.97 (0.90 - 1.05)	163	35.6%	0.84 (0.66 - 1.07)	0.212
Handwashing	1357	44.6%	0.97 (0.90 - 1.05)	139	36.0%	0.87 (0.67 - 1.14)	0.408
Nutrition	1329	43.1%	0.92 (0.84 - 1.01)	143	37.8%	0.87 (0.66 - 1.14)	0.689
WSH	1415	45.3%	0.97 (0.90 - 1.05)	148	41.2%	0.96 (0.77 - 1.19)	0.875
WSHN	1473	45.8%	0.97 (0.91 - 1.05)	184	39.7%	0.94 (0.76 - 1.15)	0.709
**Panting, wheezing or difficulty breathing**							
Control	4327	13.6%		438	10.5%		
Water	1394	14.8%	1.07 (0.90 - 1.28)	150	6.0%	0.47 (0.23 - 0.97)	0.031
Sanitation	1382	10.9%	0.83 (0.69 - 1.00)	163	11.7%	1.12 (0.64 - 1.95)	0.292
Handwashing	1357	13.3%	0.98 (0.84 - 1.14)	139	10.1%	0.99 (0.54 - 1.83)	0.952
Nutrition	1327	12.4%	0.92 (0.75 - 1.13)	143	9.8%	0.90 (0.50 - 1.60)	0.930
WSH	1415	13.6%	1.02 (0.85 - 1.23)	148	9.5%	0.77 (0.45 - 1.31)	0.327
WSHN	1473	12.6%	0.92 (0.76 - 1.11)	184	13.6%	1.22 (0.81 - 1.85)	0.155
**Fever and ARI**							
Control	4326	25.6%		438	16.4%		
Water	1394	27.8%	1.07 (0.96 - 1.20)	150	20.0%	1.26 (0.81 - 1.96)	0.511
Sanitation	1383	25.2%	0.99 (0.89 - 1.11)	163	16.0%	0.91 (0.56 - 1.48)	0.723
Handwashing	1357	24.6%	0.97 (0.86 - 1.09)	139	14.4%	0.87 (0.52 - 1.45)	0.682
Nutrition	1327	23.1%	0.89 (0.78 - 1.01)	143	14.0%	0.76 (0.45 - 1.28)	0.551
WSH	1415	25.1%	0.97 (0.88 - 1.08)	148	17.6%	1.05 (0.66 - 1.66)	0.761
WSHN	1472	25.7%	0.98 (0.88 - 1.09)	184	20.1%	1.17 (0.79 - 1.73)	0.397
**Visibly runny nose**							
Control	4300	6.8%		122	4.1%		
Water	1375	6.8%	1.05 (0.81 - 1.36)	32	0.0%	NA	NA
Sanitation	1378	6.4%	1.01 (0.77 - 1.32)	38	0.0%	NA	NA
Handwashing	1358	6.8%	0.93 (0.74 - 1.18)	39	2.6%	0.68 (0.07 - 6.23)	0.780
Nutrition	1315	7.1%	1.09 (0.85 - 1.40)	31	0.0%	NA	NA
WSH	1387	6.6%	1.04 (0.79 - 1.35)	34	5.9%	2.73 (0.46 - 16.3)	0.300
WSHN	1452	6.1%	0.90 (0.66 - 1.22)	48	0.0%	NA	NA

*An ARI was defined as caregiver-reported cough or difficulty breathing, including panting and wheezing, within the past seven days prior to the interview

Table S9: Respiratory outcome prevalence differences by index child status, interventions vs. control

Subgroup	Index Children			Non-index Children			
	**N**	**Prev.**	**Prev. Difference (95% CI)**	**N**	**Prev.**	**Prev. Difference (95% CI)**	**Interaction P-value**
**Acute respiratory infection (ARI)***							
Control	4331	46.5%		438	41.1%		
Water	1395	48.7%	0.02 (-0.02 - 0.05)	150	44.0%	0.01 (-0.09 - 0.12)	0.991
Sanitation	1383	44.9%	-0.01 (-0.5 - 0.02)	163	35.6%	-0.07 (-0.16 - 0.02)	0.203
Handwashing	1357	44.6%	-0.01 (-0.05 - 0.02)	139	36.0%	-0.05 (-0.15 - 0.05)	0.417
Nutrition	1329	43.1%	-0.04 (-0.08 - 0.00)	143	37.8%	-0.06 (-0.16 - 0.05)	0.733
WSH	1415	45.3%	-0.01 (-0.05 - 0.02)	148	41.2%	-0.02 (-0.11 - 0.07)	0.897
WSHN	1473	45.8%	-0.01 (-0.05 - 0.02)	184	39.7%	-0.03 (-0.11 - 0.05)	0.706
**Panting, wheezing or difficulty breathing**							
Control	4327	13.6%		438	10.5%		
Water	1394	14.8%	0.01 (-0.02 - 0.04)	150	6.0%	-0.06 (-0.11 - -0.01)	0.009
Sanitation	1382	10.9%	-0.02 (-0.04 - 0.00)	163	11.7%	0.01 (-0.05 - 0.08)	0.276
Handwashing	1357	13.3%	0.00 (-0.02 - 0.02)	139	10.1%	0.00 (-0.06 - 0.06)	0.909
Nutrition	1327	12.4%	-0.01 (-0.04 - 0.02)	143	9.8%	-0.01 (-0.07 - 0.05)	0.980
WSH	1415	13.6%	0.00 (-0.02 - 0.03)	148	9.5%	-0.02 (-0.07 - 0.02)	0.328
WSHN	1473	12.6%	-0.01 (-0.03 - 0.01)	184	13.6%	0.02 (-0.03 - 0.08)	0.163
**Fever and ARI**							
Control	4326	25.6%		438	16.4%		
Water	1394	27.8%	0.02 (-0.01 - 0.05)	150	20.0%	0.04 (-0.04 - 0.12)	0.276
Sanitation	1383	25.2%	0.0 (-0.03 - 0.03)	163	16.0%	-0.02 (-0.09 - 0.06)	0.639
Handwashing	1357	24.6%	-0.01 (-0.04 - 0.02)	139	14.4%	-0.02 (-0.09 - 0.05)	0.756
Nutrition	1327	23.1%	-0.03 (-0.06 - 0.00)	143	14.0%	-0.05 (-0.12 - 0.03)	0.702
WSH	1415	25.1%	-0.01 (-0.03 - 0.02)	148	17.6%	0.01 (-0.07 - 0.08)	0.759
WSHN	1472	25.7%	-0.01 (-0.03 - 0.02)	184	20.1%	0.03 (-0.05 - 0.10)	0.421
**Visibly runny nose**							
Control	4300	6.8%		122	4.1%		
Water	1375	6.8%	0.0 (-0.01 - 0.02)	32	0.0%	NA	NA
Sanitation	1378	6.4%	0.00 (-0.02 - 0.02)	38	0.0%	NA	NA
Handwashing	1358	6.8%	0.00 (-0.02 - 0.01)	39	2.6%	-0.01 (-0.07 - 0.06)	0.897
Nutrition	1315	7.1%	0.01 (-0.01 - 0.02)	31	0.0%	NA	NA
WSH	1387	6.6%	0.00 (-0.02 - 0.02)	34	5.9%	0.05 (-0.05 - 0.14)	0.378
WSHN	1452	6.1%	-0.01 (-0.03 - 0.01)	48	0.0%	NA	NA

*An ARI was defined as caregiver-reported cough or difficulty breathing, including panting and wheezing, within the past seven days prior to the interview

Table S10: Respiratory outcome prevalence differences by child sex, interventions vs. control

Subgroup	Male			Female			
	N	Prev.	Prev. Difference (95% CI)	N	Prev.	Prev. Difference (95% CI)	Interaction P-value
**Acute respiratory infection (ARI)***							
Control	2254	44.8%		2487	47.4%		
Water	724	51.0%	0.05 (0.00 - 0.10)	812	46.1%	-0.02 (-0.06 - 0.03)	0.033
Sanitation	751	42.7%	-0.02 (-0.07 - 0.03)	783	45.5%	-0.02 (-0.07 - 0.03)	0.907
Handwashing	736	45.2%	0.00 (-0.04 - 0.05)	755	42.4%	-0.04 (-0.08 - 0.01)	0.180
Nutrition	760	43.4%	-0.03 (-0.08 - 0.02)	705	42.1%	-0.05 (-0.10 - 0.01)	0.641
WSH	742	46.1%	0.01 (-0.03 - 0.06)	812	44.3%	-0.04 (-0.07 - 0.00)	0.078
WSHN	799	43.2%	-0.03 (-0.08 - 0.02)	847	47.3%	0.00 (-0.04 - 0.04)	0.318
**Panting, wheezing or difficulty breathing**							
Control	2252	13.8%		2485	13.0%		
Water	724	15.5%	0.01 (-0.03 - 0.04)	811	12.8%	0.00 (-0.03 - 0.03)	0.800
Sanitation	750	10.7%	-0.03 (-0.06 - 0.00)	783	11.5%	-0.01 (-0.04 - 0.01)	0.329
Handwashing	736	13.0%	-0.01 (-0.04 - 0.03)	755	13.0%	0.00 (-0.03 - 0.02)	0.850
Nutrition	760	10.4%	-0.04 (-0.07 - -0.01)	703	14.1%	0.02 (-0.02 - 0.06)	0.022
WSH	742	14.4%	0.01 (-0.02 - 0.04)	812	12.2%	-0.01 (-0.04 - 0.02)	0.394
WSHN	799	12.4%	-0.02 (-0.05 - 0.01)	847	13.1%	0.00 (-0.03 - 0.04)	0.290
**Fever and ARI**							
Control	2252	24.2%		2484	25.5%		
Water	724	28.0%	0.04 (0.00 - 0.08)	811	26.3%	0.01 (-0.03 - 0.04)	0.261
Sanitation	751	23.3%	-0.01 (-0.05 - 0.03)	783	25.5%	0.00 (-0.04 - 0.03)	0.794
Handwashing	736	25.8%	0.01 (-0.03 - 0.06)	755	21.7%	-0.03 (-0.07 - 0.00)	0.093
Nutrition	760	21.6%	-0.04 (-0.08 - 0.00)	703	23.0%	-0.02 (-0.07 - 0.02)	0.646
WSH	742	24.9%	0.01 (-0.03- 0.05)	812	24.1%	-0.02 (-0.05 - 0.01)	0.160
WSHN	799	25.5%	0.01 (-0.03 - 0.04)	846	24.9%	-0.01 (-0.04 - 0.02)	0.485
**Visibly runny nose**							
Control	2110	6.7%		2312	6.7%		
Water	663	5.4%	-0.01 (-0.04 - 0.01)	744	7.8%	0.02 (0.00 - 0.04)	0.025
Sanitation	685	6.4%	0.00 (-0.02 - 0.03)	731	6.0%	0.00 (-0.03 - 0.02)	0.632
Handwashing	679	6.2%	-0.01 (-0.03 - 0.01)	718	7.1%	0.00 (-0.03 - 0.02)	0.752
Nutrition	712	7.0%	0.01 (-0.01 - 0.03)	634	6.9%	0.00 (-0.02 - 0.02)	0.633
WSH	672	5.2%	-0.01 (-0.03 - 0.01)	748	7.8%	0.02 (-0.01 - 0.04)	0.028
WSHN	719	5.3%	-0.01 (-0.04 - 0.01)	781	6.4%	0.00 (-0.03 - 0.02)	0.349

*An ARI was defined as caregiver-reported cough or difficulty breathing, including panting and wheezing, within the past seven days prior to the interview

Table S11: Respiratory outcome prevalence differences by malaria seasonality, interventions vs. control

Subgroup	Malaria Season			Not Malaria Season			
	N	Prev.	Prev. Difference (95% CI)	N	Prev.	Prev. Difference (95% CI)	Interaction P-value
**Acute respiratory infection (ARI)***							
Control	1693	49.3%		3076	44.2%		
Water	521	51.2%	0.02 (-0.04 - 0.07)	1024	46.7%	0.01 (-0.03 - 0.06)	0.922
Sanitation	501	46.9%	-0.03 (-0.09 - 0.03)	1045	42.5%	-0.01 (-0.05 - 0.03)	0.677
Handwashing	525	45.0%	-0.04 (-0.09 - 0.01)	971	43.2%	-0.01 (-0.05 - 0.04)	0.362
Nutrition	553	44.3%	-0.06 (-0.12 - 0.00)	919	41.6%	-0.03 (-0.07 - 0.02)	0.382
WSH	634	42.4%	-0.07 (-0.12 - -0.02)	929	46.6%	0.02 (-0.02 - 0.06)	0.006
WSHN	615	45.9%	-0.04 (-0.09 - 0.02)	1042	44.6%	0.00 (-0.04 - 0.04)	0.298
**Panting, wheezing or difficulty breathing**							
Control	1691	14.7%		3074	12.5%		
Water	520	15.6%	0.01 (-0.04 - 0.06)	1024	13.2%	0.00 (-0.03 - 0.03)	0.754
Sanitation	500	12.8%	-0.02 (-0.04 - 0.01)	1045	10.1%	-0.02 (-0.05 - 0.01)	0.835
Handwashing	525	14.9%	0.00 (-0.03 - 0.03)	971	12.0%	-0.01 (-0.03 - 0.02)	0.791
Nutrition	552	12.3%	-0.03 (-0.06 - 0.01)	918	12.0%	0.00 (-0.04 - 0.03)	0.271
WSH	634	11.5%	-0.03 (-0.07 - 0.00)	929	14.3%	0.02 (0.00 - 0.05)	0.012
WSHN	615	14.6%	0.00 (-0.04 - 0.04)	1042	11.5%	-0.01 (-0.04 - 0.02)	0.655
**Fever and ARI**							
Control	1693	26.5%		3071	23.8%		
Water	521	29.9%	0.04 (-0.01 - 0.09)	1023	25.5%	0.01 (-0.02 - 0.04)	0.226
Sanitation	501	25.0%	-0.02 (-0.06 - 0.03)	1045	23.9%	0.00 (-0.03 - 0.04)	0.552
Handwashing	525	24.6%	-0.02 (-0.07 - 0.03)	971	23.2%	0.00 (-004 - 0.03)	0.551
Nutrition	553	23.7%	-0.04 (-0.10 - 0.01)	917	21.3%	-0.02 (-0.06 - 0.01)	0.556
WSH	634	23.2%	-0.03 (-0.07 - 0.01)	929	25.2%	0.01 (-0.02 - 0.04)	0.071
WSHN	615	25.7%	-0.02 (-0.07 - 0.03)	1041	24.8%	0.00 (-0.03 - 0.04)	0.473
**Visibly runny nose**							
Control	1444	6.0%		2978	7.1%		
Water	437	7.6%	0.02 (-0.01 - 0.05)	970	6.3%	-0.01 (-0.03 - 0.02)	0.169
Sanitation	424	9.4%	0.04 (0.00 - 0.08)	992	4.8%	-0.02 (-0.04 - 0.00)	0.012
Handwashing	465	7.1%	0.00 (-0.02 - 0.03)	932	6.4%	-0.01 (-0.03 - 0.01)	0.355
Nutrition	455	8.6%	0.03 (-0.01 - 0.07)	891	6.2%	-0.01 (-0.03 - 0.01)	0.104
WSH	541	7.0%	0.02 (-0.02 - 0.05)	880	6.3%	0.00 (-0.02 - 0.02)	0.324
WSHN	508	6.1%	0.01 (-0.03 - 0.04)	992	5.7%	-0.01 (-0.04 - 0.01)	0.325

*An ARI was defined as caregiver-reported cough or difficulty breathing, including panting and wheezing, within the past seven days prior to the interview

Table S12: Prevalence of breastfeeding indicators

Outcome, Arm	N	Prev.
**Any breastfeeding in past 24 hours, year one**		
Control	1184	78.0%
Water	535	80.8%
Sanitation	540	76.7%
Handwashing	542	81.2%
Nutrition	531	76.8%
WSH	582	81.3%
WSHN	559	78.4%
**Any breastfeeding in past 24 hours, year two**		
Control	1541	28.8%
Water	703	31.0%
Sanitation	730	27.8%
Handwashing	698	31.2%
Nutrition	669	31.5%
WSH	693	29.7%
WSHN	747	31.9%
**Early initiation of breastfeeding**		
Control	1499	52.3%
Water	693	51.5%
Sanitation	692	53.6%
Handwashing	695	48.2%
Nutrition	678	78.3%
WSH	729	50.2%
WSHN	744	76.5%
**Exclusive breastfeeding for 6 months**		
Control	1495	32.4%
Water	691	38.8%
Sanitation	688	39.7%
Handwashing	694	36.3%
Nutrition	676	56.1%
WSH	727	30.8%
WSHN	742	50.5%

Table S13: Prevalence of breastfeeding indicators, stratified by child sex

	Male		Female	
**Outcome**	**N**	**Prev.**	**N**	**Prev.**
Any breastfeeding in past 24 hours, year one	2097	81.8%	2196	82.6%
Any breastfeeding in past 24 hours, year two	2604	32.7%	2720	32.5%
Early initiation of breastfeeding	2803	56.9%	2927	58.7%
Exclusive breastfeeding for 6 months	2793	39.7%	2920	39.3%

Table S14: Unadjusted effects of interventions on prevalence of reported runny nose, intervention vs. control

Arm	N	Prev.	Prev. Ratio (95% CI)	P-value	Prev. Difference (95% CI)	P-value
Control	4769	60.5%				
Water	1545	59.9%	0.99 (0.94 - 1.04)	0.611	-0.01 (-0.04 - 0.02)	0.610
Sanitation	1546	61.9%	1.04 (98.9 - 1.09)	0.133	0.02 (-0.01 - 0.05)	0.138
Handwashing	1497	58.3%	0.98 (0.93 - 1.03)	0.335	-0.01 (-0.04 - 0.01)	0.330
Nutrition	1472	57.1%	0.94 (0.88 - 1.00)	0.045	-0.04 (-0.07 - 0.00)	0.041
WSH	1563	59.4%	0.98 (0.93 - 1.04)	0.576	-0.01 (-0.04 - 0.02)	0.574
WSHN	1658	59.3%	0.98 (0.93 - 1.02)	0.334	-0.01 (-0.04 - 0.01)	0.332

Table S15: Reported runny nose prevalence ratios and differences by malaria seasonality interventions vs. control

Subgroup	Malaria Season				Not Malaria Season				
	N	Prev.	Prev. Ratio (95% CI)	Prev. Difference (95% CI)	N	Prev.	Prev. Ratio (95% CI)	Prev. Difference (95% CI)	Interaction P-value
Control	1693	64.3%			3076	58.4%			
Water	521	61.4%	0.96 (0.90 - 1.02)	-0.03 (-0.07 - 0.01)	1024	59.2%	1.00 (0.94 - 1.07)	0.00 (-0.04 - 0.04)	0.313
Sanitation	501	65.3%	1.02 (0.93 - 1.12)	0.01 (-0.05 - 0.08)	1045	60.3%	1.05 (0.99 - 1.11)	0.03 (0.00 - 0.06)	0.654
Handwashing	526	57.4%	0.91 (0.83 - 0.99)	-0.06 (-0.11 - -0.01)	971	58.8%	1.02 (0.95 - 1.09)	0.01 (-0.03 - 0.05)	0.062
Nutrition	553	58.8%	0.92 (0.84 - 1.00)	-0.05 (-0.11 - 0.00)	919	56.0%	0.95 (0.88 - 1.03)	-0.03 (-0.07 - 0.02)	0.520
WSH	634	59.3%	0.93 (0.86 - 1.01)	-0.04 (-0.09 - 0.01)	929	59.4%	1.02 (0.95 - 1.10)	0.01 (-0.03 - 0.05)	0.112
WSHN	615	62.1%	0.96 (0.89 - 1.04)	-0.02 (-0.07 - 0.03)	1043	57.6%	0.98 (0.92 - 1.05)	-0.01 (-0.05 - 0.03)	0.718

Interaction P-value corresponds to prevalence ratio.

Figure S1: Acute respiratory infections by calendar month

Individual children were measured once at each round of follow-up, but due to the large study size each follow-up measurement spanned approximately one calendar year. Control and intervention clusters were geographically matched, and matched clusters were measured concurrently. All intervention arms had similar prevalence during follow-up (Figure 2) and were combined into a single data series in this figure to have sufficient observations to estimate monthly prevalence. The control data series has a median of 213 observations per month (first quartile (Q1): 193, third quartile (Q3): 267) and the intervention data series has a median of 418 observations per month (Q1: 337, Q3: 507).

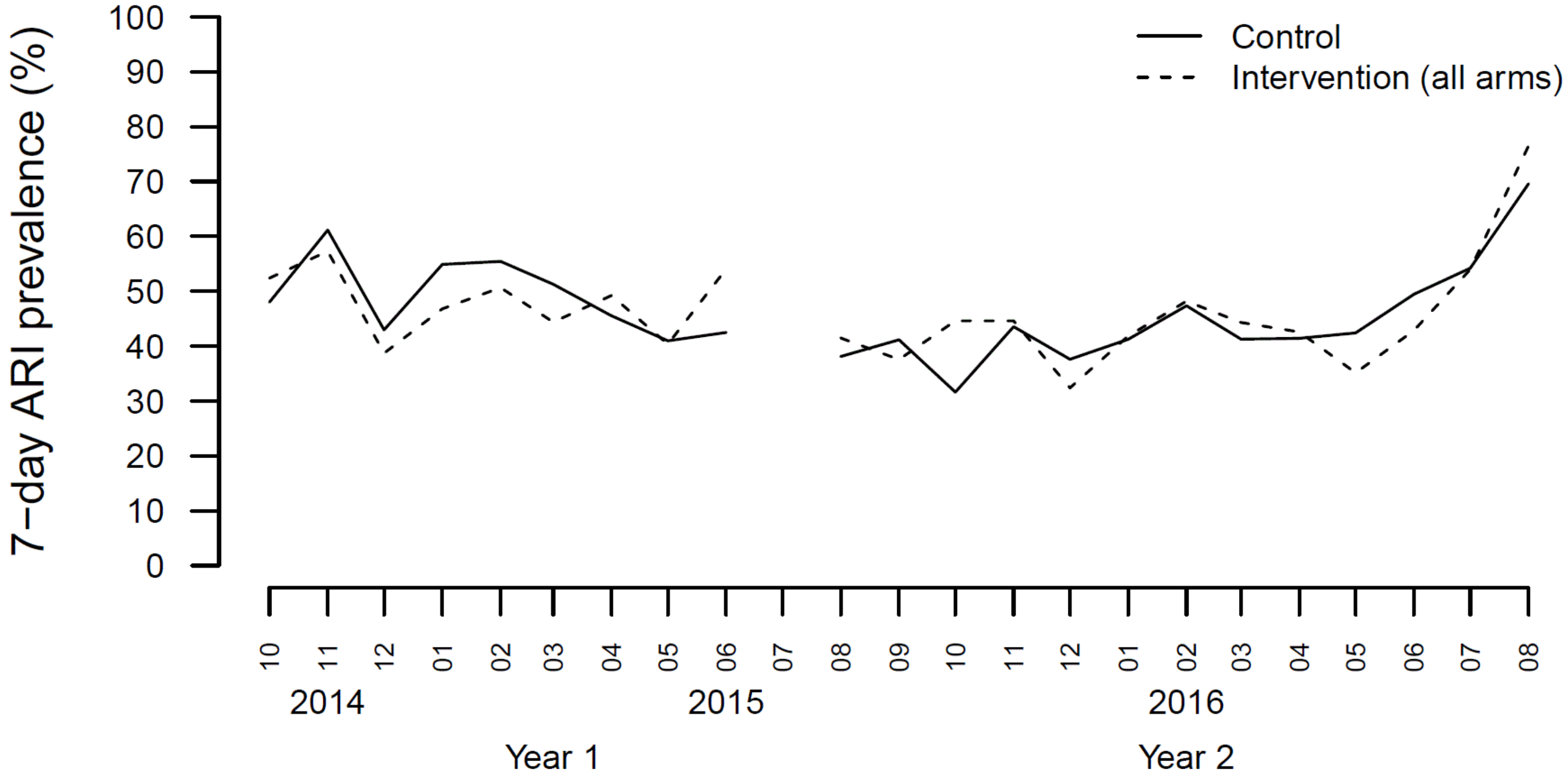

Supplement: Supplementary file 1 [file tpmd190779.SD1.pdf]
